# Novel paracellular marker based on ᴅ-dipeptide structure for highly sensitive quantification with UPLC-MS/MS for in vitro and in vivo blood-brain barrier permeability analysis

**DOI:** 10.1186/s12987-026-00806-5

**Published:** 2026-04-10

**Authors:** Cindy Bay, Eric Mühlberg, Philipp Uhl, Julia Carolin Stingl, Gzona Bajraktari-Sylejmani, Jürgen Burhenne, Johanna Weiss, Max Sauter

**Affiliations:** 1 Department of Clinical Pharmacology and Pharmacoepidemiology, Internal Medicine IX, Heidelberg University, Medical Faculty Heidelberg / Heidelberg University Hospital, Im Neuenheimer Feld 410, 69120 Heidelberg, Germany; 2 Department of Nuclear Medicine, Heidelberg University, Medical Faculty Heidelberg / Heidelberg University Hospital, Im Neuenheimer Feld 400, 69120 Heidelberg, Germany; 3 Department of Pharmaceutical Technology and Biopharmacy, Institute for Pharmacy and Molecular Biotechnology, Heidelberg University, Im Neuenheimer Feld 329, 69120 Heidelberg, Germany

**Keywords:** BCEC, Blood-brain barrier, LAT-1, Paracellular marker, PepT1, Permeability, Tandem mass spectrometry, UPLC

## Abstract

**Background:**

The blood-brain barrier (BBB) paracellular permeability must be evaluated in various contexts in vitro and in vivo, including pharmacological evaluation of drug candidates, investigations of pathological changes in disease and model development. However, most available paracellular marker substances are either radioactive, lack sensitivity and thereby require large sample volumes, or they can influence the BBB themselves via osmotic pressure. Moreover, in drug permeability studies, an adequate paracellular marker should be detectable in the same sample as the compound being tested, ideally applying the same analytical technology.

**Methods:**

We rationally designed a novel permeability marker to be highly sensitively measurable with ultra-performance liquid chromatography tandem mass spectrometry (UPLC-MS/MS); to be not a potential substrate of solute carriers or transporters; stable against enzymatic digestion and well tolerated. We evaluated this novel permeability marker in vitro in Transwell^®^ models with four different cell types (MDCK II, hCMEC/D3, primary and from induced pluripotent stem cells derived brain capillary endothelial cells) and cross-validated these results with known fluorescence markers. We further analyzed its in vivo pharmacokinetics and brain-to-plasma ratio in healthy Swiss mice. Possible interactions with LAT-1 and PepT1 were evaluated with uptake and/or inhibition assays.

**Results:**

Based on non-canonical and ᴅ-dipeptide structure, the developed marker ᴅ-methyl-tyrosinyl-ᴅ-ornithine (ᴅ-Tyr(Me)Orn) is hydrophilic and with low molecular weight (309 Da), it enables a low endogenous background and is readily available through peptide synthesis, also as isotopically labeled derivative. The developed UPLC-MS/MS quantification assay allows a highly sensitive measurement in different matrices (lower limit of quantification: 0.05 ng/mL cell medium, lysed cells, 0.1 ng/mL mouse plasma; 3 ng/g, mouse brain). In the well-known MDCK II Transwell^®^ model, it revealed a suitable P_app_ of 5.13 ± 1.08 × 10^− 7^ cm/s. Using the marker ᴅ-Tyr(Me)Orn it was further possible to compare the tightness of three brain capillary endothelial cell models. The time dependent distribution and in vivo pharmacokinetics was determined in healthy Swiss mice and revealed a constantly low brain concentration and brain-to-plasma ratio.

**Conclusion:**

We developed a highly feasible new paracellular marker, easily adaptable in various experimental designs.

**Graphical Abstract:**

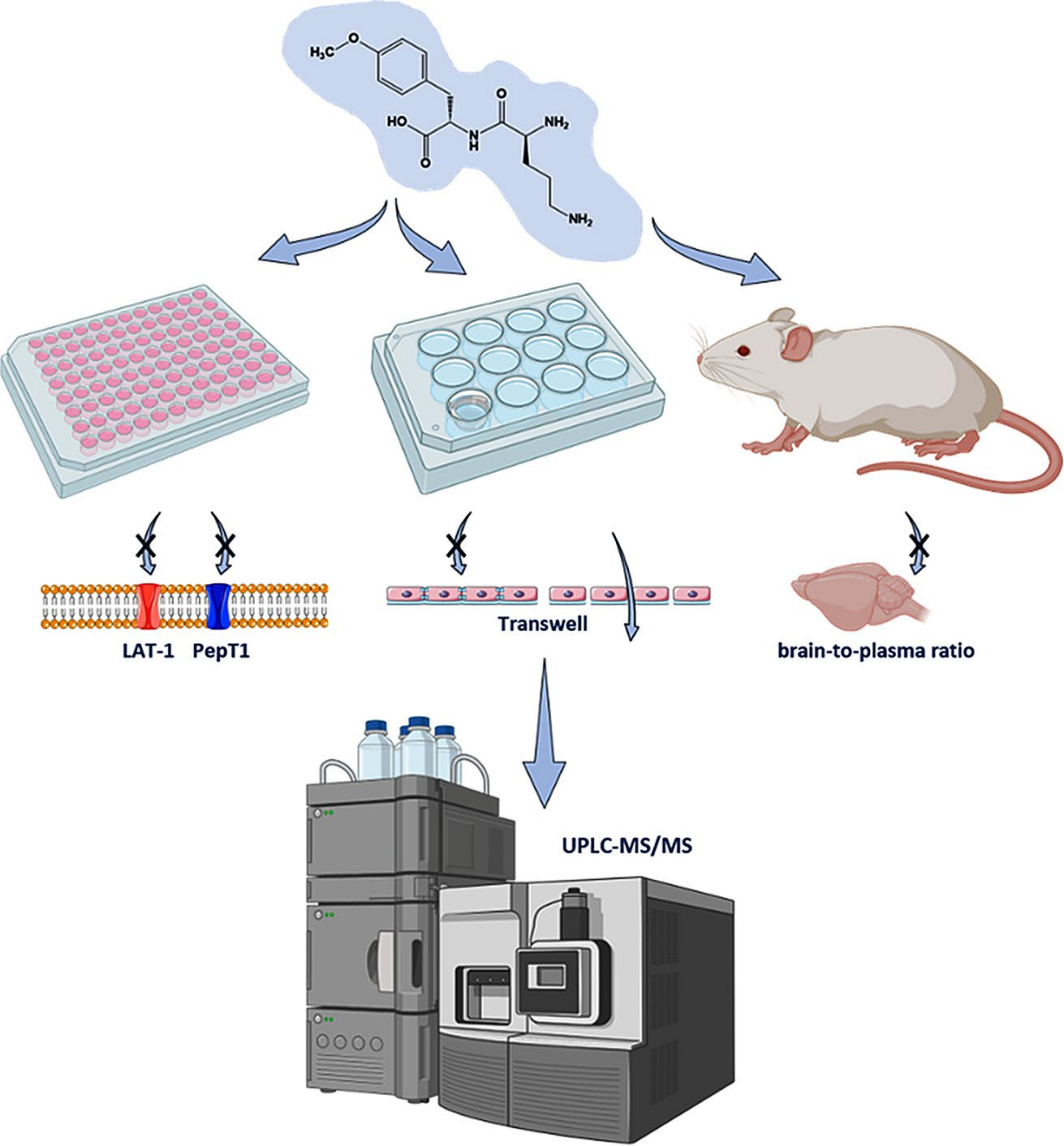

**Supplementary Information:**

The online version contains supplementary material available at 10.1186/s12987-026-00806-5.

## Background

The paracellular flux of substances is highly regulated in most physiological barriers with changes occurring in different states of disease. Considerable effort has gone into developing cell models of many barriers with the blood-brain barrier (BBB) - the most selective and restrictive barrier - receiving primary attention. Knowledge of drug permeation and hence, accessibility to the central nervous system (CNS), is an important hallmark of drug development, with access warranted for candidates acting on the CNS or to prevent side effects for other indications. Furthermore, paracellular permeability can be enhanced by various pathological conditions when brain capillary endothelial tight junctions (TJs) are impaired in vivo, so it is important to assess this damage in many contexts [1–4]. Additionally, modulation of TJ integrity is a popular approach for delivering impermeable drugs across barriers [2, 5–8]. Therefore, a reliable assessment of paracellular flux as a surrogate for TJ (dis)integrity is essential for investigating pathological changes and the prediction of the permeability of substances across physiological barriers. In particular, it is necessary for the evaluation of the relevance and translatability of in vitro models.

The conventional method for assessing this permeability is the measurement of the transendothelial electrical resistance (TEER), which depends on the free paracellular ion diffusion. However, this method is laborious and not easily applicable in vivo or in more complicated cell culture models, such as 3D-organoids. Additionally, these measurements can only be performed before and after the permeability tests hampering their applicability as internal controls and reducing the throughput capacity of such experiments. This is, however, required for efficient screenings. Paracellular marker substances can be used to easily control paracellular flux capacity and as internal controls [9].

The essential characteristics of paracellular markers are (1) the lack of permeability across lipid bilayers usually fostered by hydrophilicity and small molecular weight, (2) non-substrates of relevant solute carriers and transporters, (3) tolerability and non-toxicity at relatively high concentrations together with highly sensitive detection methods to enable the measurement of minimal amounts in acceptor compartments or tissue.

A simple strategy for paracellular marker substances is to use dyes that can be measured in high-throughput formats and enable sensitive detection. The BBB was historically discovered using such a dye, trypan blue (961 Da), between 1885 and 1913 [10, 11]. Since then, fluorescent dyes with lower protein binding have been developed like fluorescein (376 Da) [2, 12]. However, due to their often not entirely polar structures, fluorescent dyes might show some degree of membrane permeability and some are substrates of active transport [2, 13]. A widely used alternative are polysaccharides like inulin (5200 Da) and dextrans coupled with fluorescent dyes, most commonly fluorescein isothiocyanate (FITC)-dextrans [12]. These polysaccharides are highly hydrophilic and inert, and can be tailored to various hydrodynamic radii by using different molecular weight (MW). They are therefore often used for size-dependent evaluations of permeability [14]. However, due to their large molecular mass (usually at least several kDa) these markers can only detect severe paracellular openings, potentially missing subtle but already relevant changes that are important to be evaluated in vivo or in vitro [15, 16].

Finally, mono- and disaccharides are also used as paracellular markers. The monosaccharide mannitol (182 Da), as well as the disaccharide sucrose (342 Da) are commonly used [17–19]. Both saccharides are not substrates of solute carriers or glucose transporters, they are very small and highly hydrophilic. This makes them ideal markers for paracellular flux. However, highly sensitive detection of carbohydrates usually requires labelling due to associated challenges for mass spectrometric detection, such as low ionization efficiency and high endogenous background by endogenous isomers [20]. Furthermore, adding fluorescent dyes to mono- and disaccharides results in a more lipophilic and larger molecule that may exhibit increased membrane permeability. Consequently, mono- and disaccharides are usually detected via radioactive labels, which entails significant regulatory and technical challenges, as well as intrinsic safety concerns. Nevertheless, they are widely used in Transwell^®^ experiments. Despite these challenges, UPLC-MS/MS methods for the quantification of mannitol and sucrose for the assessment of permeability in vitro and in vivo were recently published with a lower limit of quantification (LLOQ) of 10 ng/mL in plasma and 100 ng/g in brain [17]; and 10 ng/g in brain [21]. However, saccharides themselves can impair the barrier integrity at high concentration due to the osmotic pressure they exert. This phenomenon is in fact being investigated for BBB opening [5, 7].

In summary, the currently used paracellular marker tracers pose limitations for label-free and highly sensitive quantification, which is required when barrier integrity and drug candidate permeability are to be assessed simultaneously. Moreover, the large number of different permeability markers hinders the comparison of results between experiments, laboratories, cell- and animal models. Therefore, our aim was to develop a novel non-toxic, non-labeled paracellular flux marker for BBB permeability assessment, together with a highly sensitive UPLC-MS/MS quantification assay. The applicability of the novel marker was evaluated in the well-known MDCK II Transwell^®^ model, controlled with cell junction modulator, and used to compare three different brain capillary endothelial cell (BCEC) types. To evaluate the in vivo use of the novel marker, its biodistribution and pharmacokinetics including the brain-to-plasma ratio in mice was determined.

## Methods

### Materials

hCMEC/D3 were purchased from Cellutions Biosystems Inc. (Burlington, Canada). Primary human brain capillary endothelial cells and Endothelial Cell Medium kit (ECM) were purchased from Innoprot (Bizkaia, Spain). Tempo-iBMEC™ human induced pluripotent stem cell (iPS)-derived Brain Microvascular Endothelial Cells (iBCEC) and the respective Maintenance Medium were obtained from Tempo Bioscience (San Francisco, CA, USA). B-27™ and human endothelial serum free medium for B27 medium, Geltrex™ LDEV-free reduced growth factor basement membrane matrix, StemPro™Accutase™ (Gibco), natrium chloride (NaCl) and the Rapid Equilibrium Dialysis (RED) Device System with inserts were purchased from Thermo Fisher Scientific (Darmstadt, Deutschland). Human collagen type IV, human fibronectin, Dulbecco’s modified Eagle’s Medium (DMEM), non-essential amino acid solution (NEAS), penicillin-streptomycin solution, L-glutamine solution, fetal bovine serum (FBS), Dulbecco’s phosphate-buffered saline (PBS), Hanks Balanced Salt Solution (HBSS), trypsin-EDTA solution (0.5 g porcine trypsin and 0.2 g EDTA/L), 1 M HEPES buffer, glycil-sarcosine (GlySar), ethyl acetate (EtOAc), ethanol (EtOH), diethyl ether, sodium bicarbonate (NaHCO_3_), methanol (MeOH), dichloromethane (DCM), Triton™ X-100 and fluorescein were purchased from Sigma Aldrich (Taufkirchen, Germany). Endothelial Growth Medium 2 SingleQuots™ was provided by Lonza (Basel, Switzerland), and Roswell Park Memorial Institute (RPMI) 1640 from PAN Biotech (Aidenbach, Germany). Laminin and ethylene glycol tetraacetic acid (EGTA) were purchased from Santa Cruz Biotechnology (Dallas, TX, USA). Standard cell culture flasks and Cell+ flasks with vented cap were obtained from Sarstedt (Nürnberg, Germany). Transwell^®^ 12 and 24 well plates were from Corning Costar (0.4 μm pore size). Human-albumin (20%, low-salt) was purchased from CSL Behring, Mouse plasma (CD-1 LiHep gender pooled) from BioIVT and bovine albumin fraction V (BSA) from AppliChem (Darmstadt, Germany). Glass beads, [^2^H_3_]-iodomethane (IC[^2^H_3_] 99.5% Atom% [^2^H_3_]) were obtained from Carl Roth (Karlsruhe, Germany). Boc-ᴅ-Tyr-OMe (96%), Fmoc N-hydroxysuccinimide ester (Fmoc-OSu), and 2-(1 H-benzotriazol-1-yl)-1,1,3,3-tetramethyluronium hexafluorophosphate (HBTU) from Carbolution (St. Ingbert, Germany). The 2 mL bead kit (2.4 mm metal beads in 2 mL reinforced microtubes) was purchased from Omni international (Kennesaw, GA, USA). Sodium sulfate (Na_2_SO_4_), sodium hydroxide (NaOH), and hydrochloric acid (HCl) were purchased from Honeywell (Seelze, Germany). Dioxane came from VWR Chemicals (Radnor, PA, USA), piperidine, diisopropylethylamine (DIEA) and trifluoroacetic acid (TFA) from Biosolve Chimie (Dieuze, France), potassium carbonate (K_2_CO_3_), from J.T. Baker (now Avantor, Radnor, PA, USA)). Dimethylformamide (DMF) and 2-chlorotrityl chloride resin were purchased from Iris Biotech GmbH. Ammonia solution (NH_4_OH) at highest analytical purity (28%) was provided by Merck (Darmstadt, Germany) and acetonitrile (ACN) and formic acid (FA) were from Biosolve (Valkenswaard, The Netherlands). Arium^®^ mini (Sartorius, Göttingen, Germany) ultrapure water system was used to produce ultra-purified water. Impact^®^ protein precipitation plates were purchased from Phenomenex (Torrance, CA, USA). Lucifer Yellow CH dipotassium salt was purchased from MCE MedChem Express (Monmouth Junction, NJ, USA). [^13^C_3_]-GlySar was prepared previously [22].

### Synthesis of ᴅ-Tyr(Me)Orn and stable isotopically labeled ᴅ-Tyr([^2^H_3_]Me)Orn

#### Synthesis of Fmoc-ᴅ-Tyr(C[^2^H_3_])-OH

Boc-ᴅ-Tyr-OMe was dissolved in DMF at a concentration of 0.3 M. 1.2 equivalents of K_2_CO_3_ and 1.1 equivalents of IC[^2^H_3_] were added and the reaction was stirred for 24 h at room temperature. Subsequently, DMF was evaporated in vacuo. The residue was dissolved in H_2_O and EtOAc and extracted using EtOAc (3 x). Organic phases were combined, washed with saturated aqueous NaCl, dried over Na_2_SO_4_, filtered and evaporated to dryness to afford the product (Boc-ᴅ-Tyr([^2^H_3_]Me)-OMe) as yellowish oil (97% yield).

Boc-ᴅ-Tyr([^2^H_3_]Me)-OMe was dissolved in EtOH at a concentration of 0.4 M and cooled to 4 °C. Then, 3 equivalents of aqueous NaOH (2 M) were added dropwise over 30 min. The reaction was acidified using aqueous HCl (1 M) and subsequently extracted using EtOAc (4 x). The organic phases were combined and evaporated to dryness to afford the product (Boc-ᴅ-Tyr([^2^H_3_]Me)-OH) as clear oil (98% yield).

Boc-ᴅ-Tyr([^2^H_3_]Me)-OH was dissolved in dioxane at a concentration of 1 M and 8 equivalents of HCl (4 M) in dioxane were added. The reaction was stirred for 4 h at room temperature, precipitated using diethyl ether, decanted, and washed with diethyl ether (2 ×). The residue was dried under vacuum to afford the product (H-ᴅ-Tyr([^2^H_3_]Me)-OH*HCl) (found *m/z* = 199.1182; calculated *m/z* 199.1157; ∆ = 12.8 ppm) as white solid (93% yield).

H-ᴅ-Tyr([^2^H_3_]Me)-OH*HCl was dissolved in H_2_O at a concentration of 0.5 M. Then, 1 equivalent of NaHCO_3_ and 1 equivalent of Fmoc-OSu (0.5 M in THF) were added and stirred for 1 h at room temperature. The solution was acidified using aqueous HCl (1 M), THF was evaporated and the reaction was extracted using EtOAc (4x). The organic phases were combined and evaporated to dryness to afford the product (Fmoc-ᴅ-Tyr([^2^H_3_]Me)-OH) *m/z* ([M + Na]^+^) = 443.1721; calculated *m/z* = 443.1657 g/mol; ∆ = 14.5 ppm) as clear oil (97% yield).

#### Solid phase peptide synthesis

Synthesis for ᴅ-Tyr(Me)-ᴅ-Orn (further referred to as ᴅ-Tyr(Me)Orn) and the isotopologue (ᴅ-Tyr([^2^H_3_]Me)Orn) were performed by solid phase peptide synthesis. Briefly, the synthesis was performed in a 100 µmol scale. 2-chlorotrityl chloride resin (100–200 mesh, 1% DVB) was loaded manually by incubation with 1 equivalent Fmoc-ᴅ-Tyr(Me)-OH/ Fmoc-ᴅ-Tyr([^2^H_3_]Me)-OH and 4 equivalents DIEA in DCM (50 mM) for 20 min at room temperature. The resin was subsequently capped by incubation with DIEA/MeOH/DCM (1:2:17) three times for 10 min each.

The preloaded resin was washed with DCM (3 x) and DMF (3 x). Fmoc-deprotection was performed using 3 mL of 20% piperidine in DMF for 5 min at room temperature. Then, 4 equivalents of Fmoc-ᴅ-Orn(Boc)-OH and 4 equivalents HBTU were dissolved in 3 mL DMF. Subsequently, 4.4 equivalents DIEA were added and the solution was incubated with the resin for 1 h at room temperature. The solution was discarded and the resin was washed with DMF (3 x) and DCM (3 x).

Cleavage from the resin was performed using 5 mL H_2_O/TFA (1:19) for 1 h at room temperature. The cleaving solution was evaporated to dryness to afford the crude product as yellowish powder. Purification was performed by preparative reversed phase HPLC (La Prep P110, VWR International) using a ReproSil-Pur120 C18-AQ column (5 μm, 150 × 25 mm). A linear gradient from H_2_O/ACN (98/2, v/v) + 1% TFA to H_2_O/ACN (80/20, v/v) + 1% TFA with a flow of 20 mL/min was used. Detection was performed via UV-absorption at λ = 214 nm. Clean fractions were collected, frozen, and lyophilized to afford the product ᴅ-Tyr(Me)Orn (found *m/z* = 310.1786 calculated *m/z* = 310.1761; ∆ = 7.95 ppm) and ᴅ-Tyr([^2^H_3_]Me)Orn (found *m/z* = 313.1994; calculated *m/z* = 313.1950; ∆ = 14.2 ppm) as clear crystals (30% yield).

ᴅ-Tyr(Me)Orn radiolabeling was performed using ^125^I as described previously [23]. In brief, 1 µmol ᴅ-Tyr(Me)Orn was dissolved in phosphate buffer (100 mM) at a concentration of 20 mM. 3 µL of aqueous Na^125^I (50 mM; approximately 6 MBq) and 1 equivalent of aqueous chloramine T (50 mM) were added. The reaction was shaken for 2 min at room temperature and quenched using saturated aqueous methionine. ^125^I-ᴅ-Tyr(Me)Orn was separated from free ^125^I by RP-HPLC (Merck Chromolith Performance RP-18e; 100 × 4.6 mm) using a linear gradient from H_2_O + 0.1% TFA to ACN/H_2_O (1/1, v/v) + 0.1% TFA over 5 min. Radioactive fractions were collected, analyzed and subsequently evaporated to dryness in vacuo. The dried residue (approximately 3 MBq) was dissolved using 0.9% aqueous NaCl for subsequent injections.

### Cell culture and in vitro experiments

All cells were incubated at 37 °C with 5% CO_2_. hCMEC/D3 were cultured in Endothelial Growth Medium 2 with all supplements of the EGM 2 kit (EGM) on Cell+ culture flasks. MDCK II cells (available at the American Type Culture Collection (ATCC, Manassas, VA, USA)) were grown in DMEM with 10% FBS, glutamine (2 mM), penicillin (100 U/mL) and streptomycin (100 µg/mL). For CaCo-2 cells (available at ATCC) this medium was further supplemented with 1% NEAS. Both cell lines were grown in standard vented cell culture flasks.

Primary human BCEC were cultured in Endothelial Cell Medium Cell+ culture flasks. IPS-derived BCEC (iBCEC) were cultured as described by the manufacturer with the included medium. After thawing they were seeded in 6-well coated for 1 h at 37 °C with 100 µg Geltrex™ matrix. For detachment, Accutase™ was used.

### Stock solutions and dilutions

All stock solutions were stored at -20 °C in small aliquots to avoid repeated freeze-and-thaw cycles. ᴅ-Tyr(Me)Orn was diluted in PBS for a stock solution concentration of 40 mM. Dilution for experiments were freshly prepared in the respective cell culture medium containing additional 7% of human plasma protein from human serum (Medium-HS) or HBSS with 0.01 M Hepes (HHBSS). 10 mM EGTA solution was prepared in PBS and pH was adjusted to 8.0 to facilitate solubility of the weighted EGTA powder. Before experiments, this solution was diluted 1:1 in the respective cell culture medium.

### ᴅ-Tyr(Me)Orn uptake and LAT-1 inhibition assay

96-well plates were coated with 2 µg/cm^2^ laminin and 7.5 × 10^4^ cells/cm^2^ hCMEC/D3 cells were seeded per well. Cells were grown until confluency and medium exchanged every 2–3 days. To evaluate if ᴅ-Tyr(Me)Orn can competitively inhibit and thus binds to the amino acid transporter LAT-1, inhibition assay was performed as described previously [24]. In brief, 50 µL of the respective concentration of ᴅ-Tyr(Me)Orn (0, 200, 600, 2000, 6000 µM) followed by 50 µL of 20 µM [^13^C_6_,^15^N]-leucine were added to the respective wells. This resulted in a final ᴅ-Tyr(Me)Orn concentration of 100, 300, 1000 and 3000 µM and a final [^13^C_6_,^15^N]-leucin concentration of 10 µM. After 10 min, the uptake was stopped by removing the medium and washing the cells on ice three times with ice-cold HHBSS. The intracellular [^13^C_6_,^15^N]-leucine concentration in the presence of ᴅ-Tyr(Me)Orn was normalized to the untreated cells.

To analyze the uptake of ᴅ-Tyr(Me)Orn by hCMEC/D3 cells 100 and 300 µM ᴅ-Tyr(Me)Orn were incubated for 10 and 60 min. For background control, precoated but empty wells were treated simultaneously. After either 10–60 min, the uptakes were terminated and the wells were washed on ice three times with ice-cold HHBSS. The background was subtracted from all groups and normalized to the untreated control (10 min, 100 µM). Intracellular leucine concentration was quantified in samples as previously described [24]. Intracellular ᴅ-Tyr(Me)Orn quantification is described below.

### Lucifer Yellow and fluorescein uptake

hCMEC/D3 cells were seeded in 96-well plates, and the uptake with 100 µM and 300 µM of either Lucifer Yellow or fluorescein solution in HHBSS was performed as described for ᴅ-Tyr(Me)Orn uptake. After washing, the cells were lysed with 100 µl of a 1% Triton™ X-100 solution in HHBSS. Fluorescence was measured using a SpectraMax^®^ plate reader, and the concentrations were subsequently calculated using respective standard curves in 1% Triton™ X-100 (0.001–3 µM), generated using linear regression in GraphPad Prism. The resulting concentrations were then used to calculate the percentage of fluorophore remaining per well.

### PepT1-inhibition assay

Possible PepT1 substrate characteristics of ᴅ-Tyr(Me)Orn were evaluated in the competitive substrate inhibition of PepT1 assay as published previously [22]. In brief, confluent CaCo-2 monolayers were grown in 96-well plates. Intracellular concentrations of GlySar, a well-known PepT1 substrate, were measured after incubation of the cells with either 20 µM GlySar alone or in the presence of increasing concentration of ᴅ-Tyr(Me)Orn (100–3000 µM ᴅ-Tyr(Me)Orn). The intracellular concentrations were then normalized to GlySar concentration in control cells without addition of ᴅ-Tyr(Me)Orn. Intracellular GlySar concentration was quantified in samples as previously described [22].

### Transwell^®^ experiments

Either hCEMC/D3 or MDCK II cells were seeded at a density of 5 × 10⁵ cells/well onto a Transwell^®^ filter of a 12-well plate. For hCEMC/D3 cells, filters were precoated with collagen (20 µg/cm^2^) and fibronectin (4 µg/cm^2^). For iBCEC and primary BCEC, filters of a 24-well Transwell^®^ plate were precoated with collagen (20 µg/cm^2^) and fibronectin (4 µg/cm^2^), and 0.9 × 10^5^ cells/well seeded. hCEMC/D3 and MDCK II cells were grown until confluency for 6 days. iBCEC and primary BCEC were left for at least 7 days in B27 medium and ECM, respectively. After 4 h of the pre-incubation (control with fresh medium) 500 µL for 12-well plate and 100 µL for 24-well plate of 300 µM of ᴅ-Tyr(Me)Orn in Medium-HS were added to the upper compartment. The lower compartment was filled with the respective Medium-HS, 1500 µL for 12-well plate and 600 µL for 24 well plate. The cells were further incubated at 37 °C under gentle agitation and 20 µL samples were taken from the lower compartment every 10 min for a total of 60 min. The removed medium was replaced in the lower compartment at each time point. Medium samples were diluted 20-fold with medium and stored at -20 °C until analysis.

To evaluate the permeability of Lucifer Yellow and fluorescein, hCMEC/D3 and MDCK II cells were seeded equally, and 500 µL of 300 µM solution of the fluorophore in HHBSS added. At each time point, a 100 µL sample was collected from the lower compartment and directly transferred to a 96-well plate, after which the compartment was replenished with fresh HHBSS. Fluorescence was measured using a SpectraMax^®^ plate reader, and the fluorophore concentration was calculated using a linear standard curve (0.001-10 µM; diluted in HHBSS), which was generated using linear regression in GraphPad Prism.

### In vivo experiments

The experiments in mice were performed in strict accordance with the German and European regulations for care and experimentation of laboratory animals (German Animal Protection Law and the Directive 2010/63/EU) approved by the Animal Care and Use Committee at the governmental institution Regierungspräsidium Karlsruhe (Karlsruhe, Germany; reference number 35-9185.81/G-199/21; date of approval: November 23, 2021). Healthy female SWISS mice were intravenously administered 1 mg of ᴅ-Tyr(Me)Orn (20 mg/ml in 0.9% NaCl) in the tail vein, under anesthesia with isoflurane, either unlabeled for UPLC-MS/MS analysis or radioactive labeled with ^125^I. For UPLC-MS/MS analysis, mice were sacrificed by CO_2_ inhalation after 5, 10, 20 and 40 min. Blood samples (50 µl) were taken, mice perfused with PBS and brain, liver and kidneys collected. Samples were stored at − 80 °C until analysis.

For radioactive imaging, mice were anesthetized using isoflurane and subsequently injected with approximately 1 MBq of ^125^I-ᴅ-Tyr(Me)Orn into the tail vain. Scintigraphic imaging was performed on a γ-camera (Gamma Imager, Biospace Lab, Paris, France) on proximal dorsal plane for a duration of 10 min directly after injection, 30 min, 1 h, and 2 h post injection.

### Evaluation of plasma protein binding

To determine the percentage of plasma protein binding, ᴅ-Tyr(Me)Orn was diluted in mouse plasma (10 ng/mL, 100 ng/mL and 2000 ng/mL) with a minimal solvent concentration being ensured. The Rapid Equilibrium Dialysis (RED) Device System was then used following the manufacturer’s instructions, with a plasma volume of 100 µL and a buffer chamber containing 350 µL PBS. Equilibration was performed at 37 °C for 4 h. Samples were taken from both sites and ᴅ-Tyr(Me)Orn concentrations were determined with UPLC-MS/MS as described below. Samples from the highest concentration (2,000 ng/mL) were diluted 10-fold in mouse plasma prior to measurement.

### Sample preparations and analytical method for ᴅ-Tyr(Me)Orn UPLC-MS/MS analysis

#### Method development for quantification of ᴅ-Tyr(Me)Orn

Measurements were performed on a triple-stage quadrupole mass spectrometer (Waters Xevo TQ-XS) using selected reaction monitoring (SRM) with argon collision-induced dissociation (CID) with Z-spray electrospray ionization (ESI) source in positive ion mode. ᴅ-Tyr(Me)Orn was dissolved in H_2_O/ACN (1/1, v/v) + 0.1% FA at a concentration of 1 µg/mL and directly injected into the mass spectrometer to auto-optimize the analysis parameters with IntelliStart of the MassLynx V4.2 system software (supplement S1). Four mass transitions were found for the respective [M + H]^+^ ions of ᴅ-Tyr(Me)Orn (309.17 g/mol, m/z) and ᴅ-Tyr([^2^H_3_]Me)Orn (312.19 g/mol, *m/z*), with decreasing intensities: *m/z* 310.2 → 115.0, *m/z* 310.2 → 70.0, *m/z* 310.2 → 293.1, *m/z* 310.2 → 196.0 and *m/z* 313.2 → 115.0, *m/z* 313.2 → 69.9, *m/z* 313.2 → 296.1, *m/z* 313.2 → 199.0. For both analytes the 115.0 fragment of ornithine (b_1_) was the most abundant generated fragment and therefore selected as quantification transition.

Chromatography was performed with a CORTECS^®^ UPLC^®^ HILIC column (1.6 μm, 2.1 × 50 mm) at 40 °C on an Acquity Classic UPLC^®^ (Waters, Milford, MA, USA). Injection was performed with full loop injection mode (20 µL). Aqueous eluent A consisted of H_2_O + 0.1% FA + 0.03% NH_4_OH, the mobile phase B of ACN/H_2_O (95/5, v/v) + 0.1% FA + 0.01% NH_4_OH. Gradient elution was performed at a flow rate of 0.5 mL/min as follows: starting with initial conditions of 15% of eluent A for 1.5 min, then the percentage of eluent A was increased to 98% over the course of 1 min. After an additional 1.1 min, the composition returned to the initial conditions and kept until the end of run time at 4 min. The next run started after 1 min of autosampler injection preparation, resulting in a total cycle time of 5 min. During method development, reverse phase chromatography was tested but did not result in sufficient retention. The added ammonia did reduce the background and improved the peak shape.

#### Calibration and sample preparation

For the calibration curve and quality control (QC) samples, two independent weighed amounts of ᴅ-Tyr(Me)Orn were performed in volumetric flasks and dissolved in 5 mL H_2_O/ACN (75/25, v/v) + 0.1% FA. Spike solutions were then prepared by diluting these stock solutions in H_2_O/ACN (75/25, v/v) + 0.1% FA to the sample concentrations of: 0.05, 0.1, 0.3, 1, 3, 10, 30, 100, 300 ng/mL for calibration. For QC solutions, concentrations of 0.05 (lower limit of quantification, LLOQ), 0.15 (low QC), 112.5 (mid QC) and 225 ng/mL (high QC) sample concentrations were prepared. The LLOQ for plasma and brain samples was adjusted, resulting in 0.1 (LLOQ), 0.3 (low QC for plasma, LLOQ for brain), 112.5 (mid QC) and 225 ng/mL (high QC) sample concentrations.

Internal standard working solution was prepared from a weigh-in of ᴅ-Tyr([^2^H_3_]Me)Orn, which was diluted in H_2_O/ACN (75/25, v/v) + 0.1% FA to a final concentration of 2.7 ng/mL. For system suitability test at the beginning and end of each run a reference solution containing 30 ng/mL of ᴅ-Tyr(Me)Orn and 10 ng/mL of ᴅ-Tyr([^2^H_3_]Me)Orn in ACN + 0.1% FA was used.

For sample preparation, 25 µL of study sample was spiked with 25 µL of internal standard working solution in a 96 well plate. The calibration curve and QC samples were generated by spiking 25 µL of respective matrix with 25 µL of internal standard working solution and the addition of calibration or QC solution. To account for the volume difference, 25 µL of H_2_O/ACN (75/25, v/v) + 0.1% FA was added to the study samples. Then, 100 µL of ACN + 0.1% FA were added to all wells for protein precipitation. If the matrix did not contain NH₄OH, the ACN was mixed with 1% NH₄OH and 10% H_2_O to ensure optimal peak shape. The plate was closed and vortexed for 1 min. After centrifugation for 10 min at 1000 × *g* the plate was transferred to the Sample Manager for UPLC-MS/MS quantification.

For each separate application, the matrix for the calibration and QC samples was adapted: study samples of Transwell^®^ experiments consisted of basolateral collected medium, therefore medium was used as matrix for calibration and QC samples. For uptake experiments in hCMEC/D3, cells were directly lysed with aqueous NH_4_OH (10%, 25 µL) in 96-well plates, therefore untreated wells with lysed cells were used as a matrix.

For mouse plasma samples, blank Li-Heparin mouse plasma was used as a matrix. For mouse brain study samples, brains were weighed and dissolved in PBS to a concentration of 100 mg/mL. The tissue was ruptured with glass beads (brain) or metal beads (liver, kidney) in the Bead Rupture 4 (Omni international). 25 µL of the resulting suspension was used as sample. The higher protein content in brain and plasma samples required the increase of ACN volume to 200 µL of ACN + 0.1% FA + 10% H_2_O + 1% NH_4_OH. These samples were then filtered with Impact™ protein precipitation plates and the extracts further centrifuged (10 min at 1000 × *g*). The supernatant was transferred into a fresh plate and transferred to the Sample Manager for quantification. Plasma samples were diluted in mouse plasma to achieve concentrations in the calibration range. Liver and kidney samples were homogenized as described for brain tissue and subsequently at least 100 × diluted in mouse plasma. They were then quantified by means of the mouse plasma calibration curve.

#### Method validation

The UPLC-MS/MS assay for quantification of ᴅ-Tyr(Me)Orn was validated based on the applicable recommendations of the ICH M10 guideline for bioanalytical method validation [25]. Three independent validation runs were performed to ensure linearity in the calibration range 0.05–300 ng/mL, as well as inter- and intra-run accuracy and precision for cell lysate. For transfer of the assay to each further matrix (cell medium, mouse plasma and mouse brain) one validation run was performed to demonstrate the applicability. Each run contained nine calibration points in duplicates and respective QC samples. Selectivity was verified for all matrices by measuring blank medium, cell lysate, mouse plasma, and mouse brain. Stability of the stock solutions at 5 ± 3 °C was proven for > 3 months. Similarly, the stability of plasma samples at -20 °C was verified for > 14 days. To ensure reproducible measurement the extract stability was tested 24 h after storage at 14 ± 3 °C in the autosampler. Stability in mouse plasma was tested after 2 h at 37 °C.

Internal standard normalized matrix effect was determined in triplicates for all matrices by dividing the response (ratio of analyte signal to internal standard signal) of QC solution spiked to extracted matrix with the respective QC solution in solvent [26]. Internal standard normalized recovery was calculated accordingly by dividing the response of QC samples by the response of QC samples spiked after extraction.

### Data analysis and statistical methods

The apparent permeability coefficient P_app_ [cm/s] was calculated as described in [27]. Following the formula:

$$\:{P}_{app}=\:\frac{dQ}{dt}\times\frac{1}{A\times{c}_{0}}$$

dQ/dt was determined by calculating the clearance volume c_v_ [cm^3^]:

$$\:\frac{{c}_{t}}{{c}_{I}}\times{v}_{D}={c}_{v}$$

Clearance volume at each time point was calculated from the measured concentration c_t_ in the basolateral compartment of the Transwells^®^ in relation to the initial concentration in the apical compartment c_I_ multiplied with the basolateral volume v_D_. The measured concentration was corrected for dilution during sampling as described for diffusion chambers (Harvard Apparatus, MA, USA) and the amount of crossed substance was determined by addressing the respective volume differences. GraphPad Prism software (version 10.6.1) was used to plot this volume over time. Simple linear regression was used to calculate the slope in the linear range between 0 and 60 min, which equals dQ/dt or the permeability surface area product PS [cm^3^/s]. The slope of empty wells 1/PS_f_ was used to subtract the impact of the filter from the total measured PS_t_ to calculate the PS_c_ of the monolayer:

$$\:\frac{1}{{PS}_{c}}=\frac{1}{{PS}_{t}}-\frac{1}{{PS}_{f}}$$

The permeability coefficient P_app_ [cm/s] was then determined by dividing with the filter surface area. If the filter subtraction did result in negative P_app_, these values were set to 0, based on biological plausibility. GraphPad Prism was further used for data visualization and statistical analysis. One-way ANOVA with Tukey’s multiple comparisons post hoc test was used for statistical testing between distinct groups, and two-way ANOVA for multiple subgroups. The details of these statistical tests are indicated on the respective figures and tables, with *p* ≤ 0.0001 ****, *p* ≤ 0.001 ***, *p* ≤ 0.01 **, *p* ≤ 0.05 * and a p value of > 0.05 considered as non-significant. Microsoft Excel 2010 (Mountain View, CA, USA) was used for data processing and calculations. For UPLC-MS/MS quantification the Waters TargetLynx V4.2 software (Waters, Milford, MA, USA) was used to calculate calibration curves based on 1/x^2^-weighted linear regressions of the response of ᴅ-Tyr(Me)Orn and the isotopologue ᴅ-Tyr([^2^H_3_]Me)Orn. All chemical structures were drawn with ChemDraw (v23.1.2).

Pharmacokinetic parameters were calculated by using non-compartmental methods (Phoenix WinNonlin 8.6, Certara, Princeton, New Jersey). The NCA object with IV bolus administration, sparse data option, an average dose of 1.02 mg, and linear-up-log-down interpolation was used. The half-life (t_½_) was calculated based on the final two concentrations. The hypothetical concentration at 0 min (C_0_) was calculated using log-linear back-extrapolation based on the initial two concentrations. Total body clearance (CL) was calculated by dividing the dose by the area under the curve extrapolated to infinity (AUC_∞_), and steady state volume of distribution (V_SS_) as CL multiplied with the mean residence time.

## Results

### Rational design of a novel ᴅ-dipeptide based BBB permeability marker suitable for mass spectrometry quantification

**Fig. 1:**
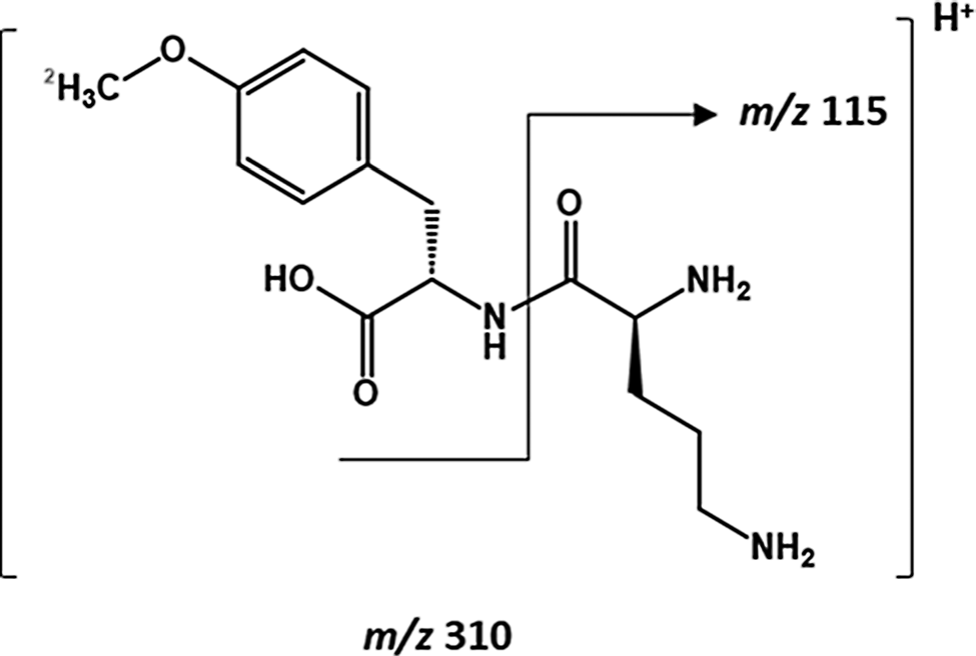
Chemical structure of the ᴅ-amino acid-based dipeptide ᴅ-Tyr(Me)Orn and its monitored mass transition. Internal standard was stable isotopically labeled at the methyl-group with [^2^H_3_] (depicted in grey)

We aimed to design a molecule that is based on nearly endogenous structure, well tolerated, while being stable, not a substrate of active transport and highly sensitively measurable with UPLC-MS/MS with low endogenous background. Based on these criteria we set on a dipeptide backbone. We selected ornithine and methylated tyrosine as suitable non-canonical ᴅ-amino acids for our novel permeability marker ᴅ-methyl-tyrosinyl-ᴅ-ornithine (Fig. 1). These amino acids are readily available. The methyl-group can easily be isotopically labeled with [^2^H_3_], which enables the independent synthesis of stable isotopically labeled internal standard required for reliable UPLC-MS/MS quantification. After solid phase synthesis and purification we achieved a yield of 30% final product ᴅ-Tyr(Me)Orn.

As intended, the dipeptide showed a high ion yield and readily fragmented at the peptide-bond. It showed a good retention on HILIC column, which could be improved with the addition of ammonia to the samples and the eluents. Overall, this enabled the limit of quantification to be set to 0.05 ng/mL for lysed cells and medium, 0.1 ng/mL for plasma, and 3 ng/g (corresponding to 0.3 ng/mL homogenate) for brain. We validated the analytical assay for ᴅ-Tyr(Me)Orn following the applicable recommendations of the guideline for bioanalytical method validation, ICH M10 [25], in cell culture medium, lysed cells, mouse plasma and mouse brain homogenates. The calibration ranges from 0.05 (0.1/0.3) – 300 ng/ml were validated for all used matrices and the linearity of the assay was always (r^2^) > 0.98. The respective parameters of the validations are given in supplement Table S2. Accuracy for all matrices was between 85.7 and 112.8% and precisions ≤ 11.5%. The consistent matrix effect and recovery for all matrices are given in supplement Table S3. The internal standard was suitable for normalization and did not show any interference signal in the monitored analyte mass transition. The design of the dipeptide with two non-canonical amino acids resulted in an acceptable endogenous background. Only the plasma and brain homogenates showed relevant background signal, which required to set the lower limit of quantification to 0.1 ng/mL for plasma and 0.3 ng/mL for brain homogenate (resulting in 3 ng/g). There was no detectable carry-over after the highest calibration point. Stability was demonstrated for at least 2 h at 37 °C in mouse plasma (accuracy: 98.4-104.6% / precision: 0.45–6.33%), proving its stability against enzymatic hydrolysis by plasma proteins. Further, plasma sample stability for storage at − 20 °C for at least 14 days was given (99.3–96.0%/ 2.46–3.73%) as well as extract stability in the autosampler for 24 h (106.8–113.1%/ 0.71–3.8%) and stock solution stability > 3 months (90.7 -109.1% / 4.17–8.80%). Representative chromatograms are shown in Figure S4.

### ᴅ-Tyr(Me)Orn is not a substrate of PepT1 and LAT-1

**Fig. 2:**
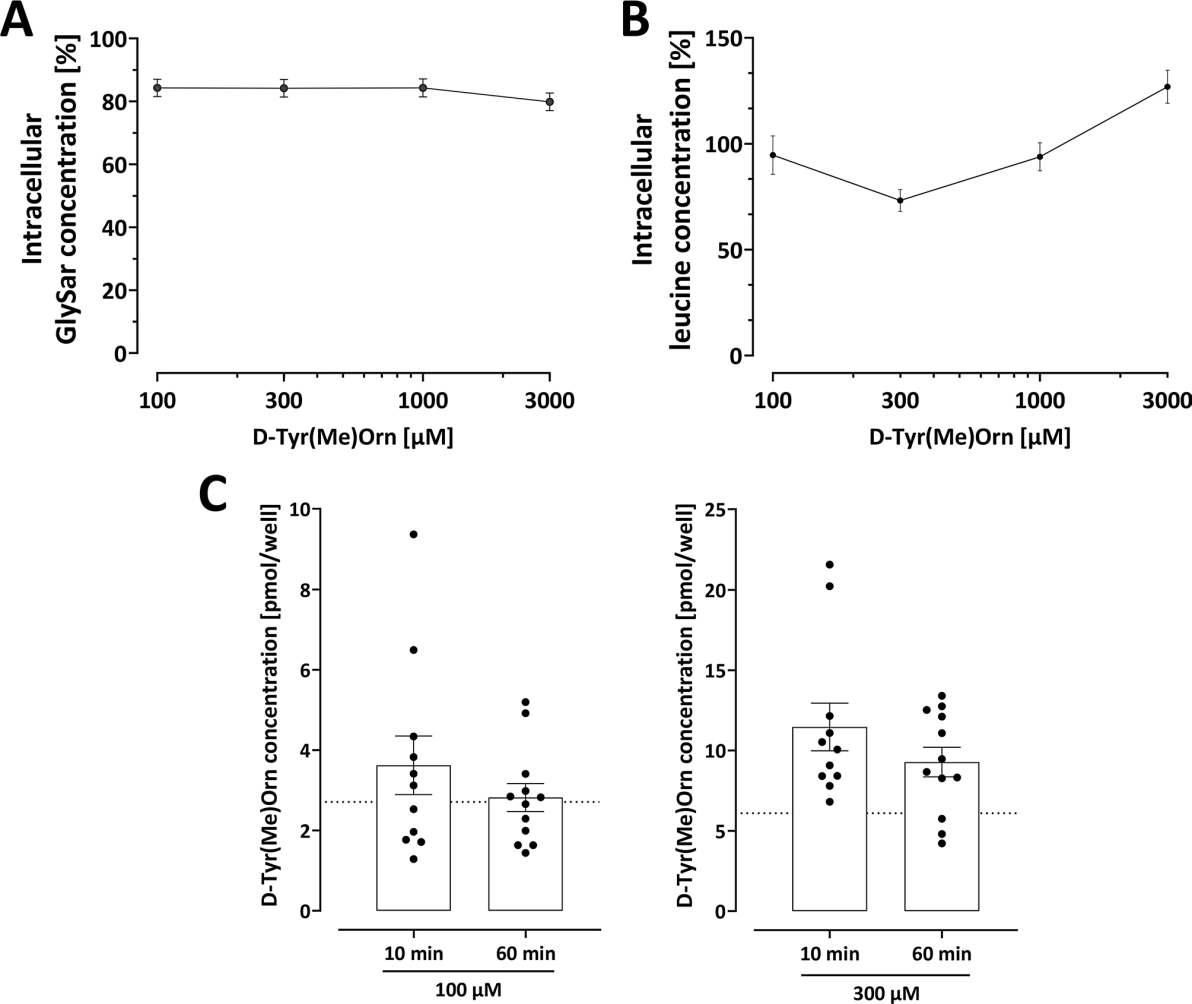
**A** ᴅ-Tyr(Me)Orn does not inhibit the intracellular uptake of glycyl-sarcosine (GlySar) in CaCo-2 cells, and is therefore no substrate of PepT1. **B** ᴅ-Tyr(Me)Orn does not inhibit the intracellular uptake of leucine in hCMEC/D3 cells, and does therefore not interact with LAT-1. **C** Only low amounts of ᴅ-Tyr(Me)Orn remained in the wells after washing, indicating no substantial uptake of ᴅ-Tyr(Me)Orn in hCMEC/D3. The dotted lines indicate the mean background. All graphs show means ± SEM from three independent experiments. There was no significant inhibition of the uptake of GlySar or leucine between 100 to 3000 µM ᴅ-Tyr(Me)Orn in **A** and **B** (one-way ANOVA with Dunnett’s multiple comparison test) and between the two time points in C (unpaired t-test)

To confirm that ᴅ-Tyr(Me)Orn is indeed not a substrate of transporters that can transport dipeptides, we evaluated first whether ᴅ-Tyr(Me)Orn interacts with PepT1 and LAT-1. In CaCo-2 cells, cellular GlySar uptake was not inhibited by increasing concentrations of ᴅ-Tyr(Me)Orn (Fig. 2, A) indicating lack of PepT1 substrate characteristics.

Secondly, the possible inhibition of LAT-1 uptake of leucine by competitive inhibition of ᴅ-Tyr(Me)Orn was tested on hCEMC/D3 monolayer. No inhibition was seen for increasing concentration of ᴅ-Tyr(Me)Orn (Fig. 2, B). These data do indicate that ᴅ-Tyr(Me)Orn is not a substrate of LAT-1.

Finally, we tested whether ᴅ-Tyr(Me)Orn can penetrate into hCMEC/D3. Only very small amounts of ᴅ-Tyr(Me)Orn remained in the wells after washing (Fig. 2, C). Following incubation at concentrations of 100 µM (10 nmol/well) and 300 µM (30 nmol/well), the remaining percentage were 0.03 ± 0.02% (3.20 ± 1.85 pmol/well) and 0.03 ± 0.01% (10.3 ± 4.07 pmol/well), respectively. Where the mean background measured in empty wells of the two concentrations alone accounted for 2.71 pmol/well and 6.10 pmol/well (dotted line in Fig. 2, C).

We performed an identical uptake experiment using the classical permeability markers Lucifer Yellow and fluorescein. After washing, comparable amounts of Lucifer Yellow (> 0.053%) and fluorescein (> 0.108%) remained in the wells of the uptake solutions (see Figure S5), confirming negligible uptake of ᴅ-Tyr(Me)Orn.

To enable comparison with active transport, in a similar format we previously measured 2.17 ± 0.54% (21.7 ± 5.4 pmol/well) leucine after 10 min incubation with 10 µM [24]. In this study, CaCo-2 cells took up 36.2 ± 11.0 pmol/well of GlySar, accounting for 4.53 ± 1.37% of the total amount incubated (Fig. 2A).

### ᴅ-Tyr(Me)Orn is a paracellular marker substance with minimal P_app_ across MDCK II monolayers

**Fig. 3:**
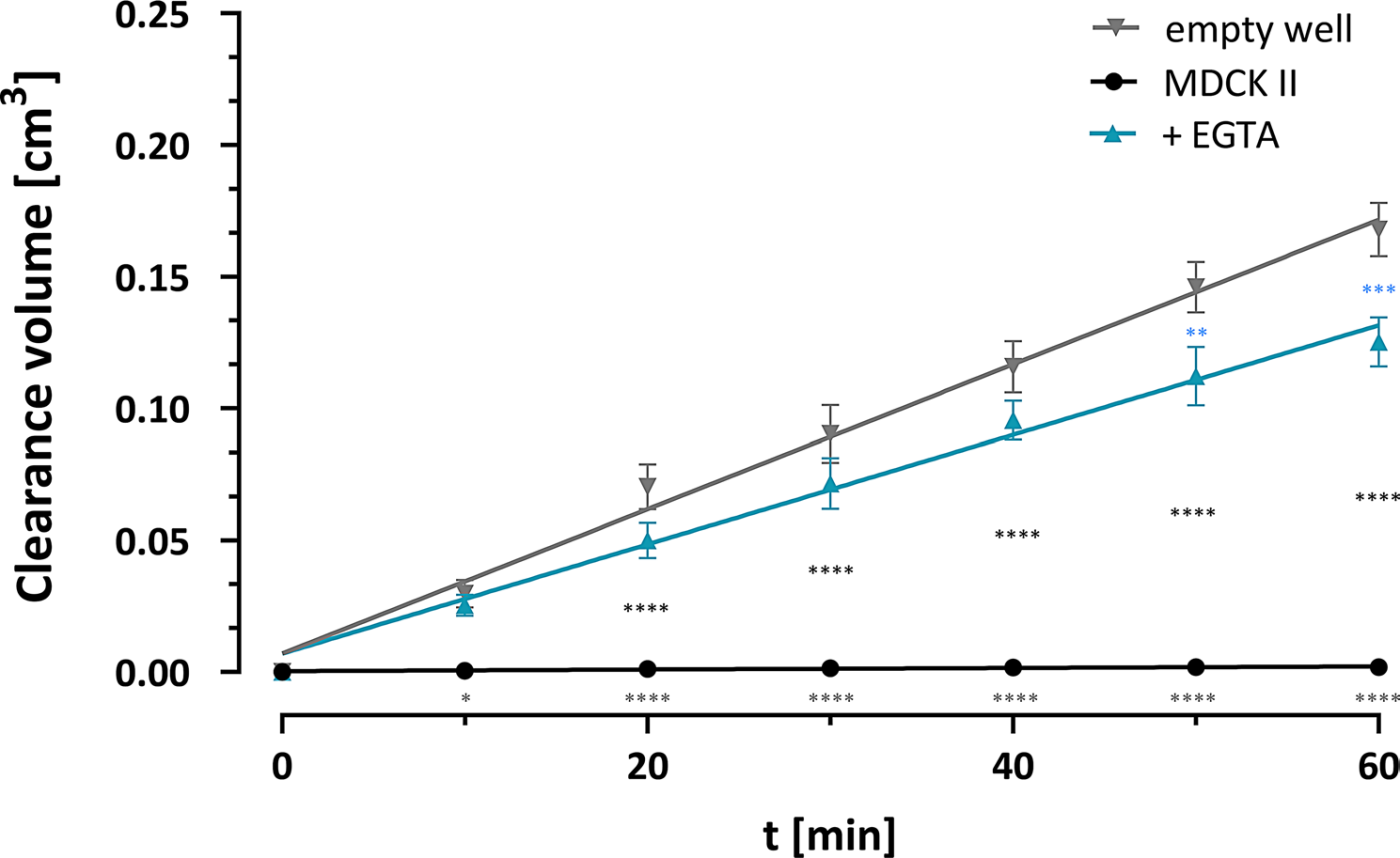
Permeability of ᴅ-Tyr(Me)Orn, shown as clearance volume over time from apical to basolateral compartment of Transwells^®^ either in empty wells, wells seeded with confluent MDCK II cells with or without 4 h EGTA preincubation (5 mM). Data represents at least seven replicates from three independent experiments, mean ± SEM. Simple linear regression was performed and the resulting slopes were used to calculate the permeability coefficient P_app_ as given in Table 1. Statistical evaluation was performed with two-way ANOVA and significances between groups at each time point are depicted in the graph (from top to bottom: blue = empty wells vs. EGTA; black = untreated MDCK II vs. EGTA; grey = empty well vs. MDCK II). The in-detail list of statistical results is shown in Table S6

To evaluate the potential use as permeability marker, the apparent permeability coefficient P_app_ was calculated in the well-known parenteral MDCK II Transwell^®^ model (Fig. 3). This resulted in a low P_app_ of 5.13 ± 1.08 × 10^− 7^ cm/s (Table 1). To test if the effect was due to the tight cell-cell connections and therefore true paracellular tightness, calcium-dependent adherens junctions were impaired with the use of EGTA. This pre-treatment increased the permeability a thousand-fold to a similar range than the filter (empty well) alone, reflected in the respective P_app_ values.

### Permeability comparison of brain capillary endothelial cell monolayer models

**Fig. 4:**
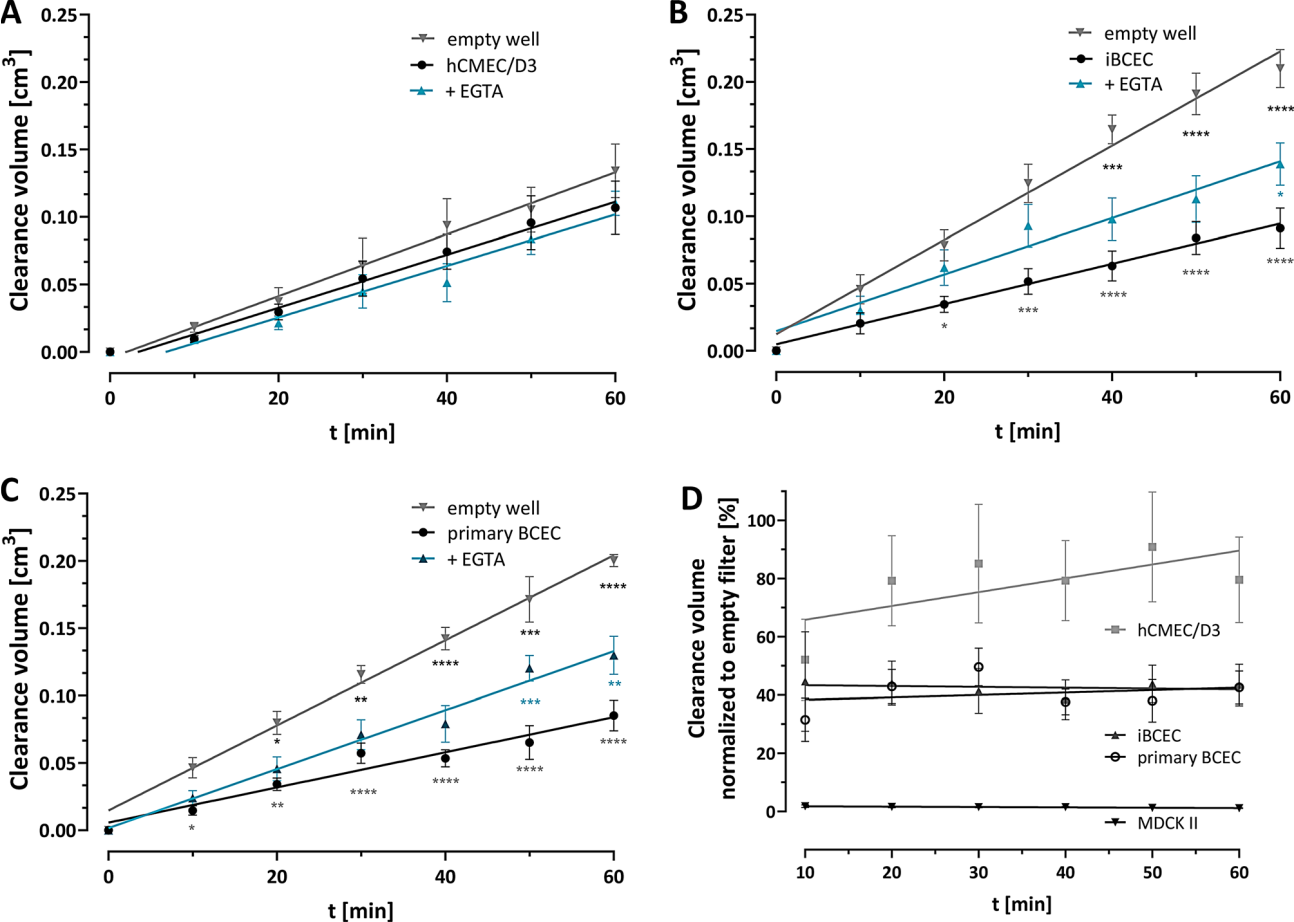
Permeability of ᴅ-Tyr(Me)Orn, shown as clearance volume over time from apical to basolateral compartment of Transwells^®^ either in empty wells, wells seeded with confluent **A** hCMEC/D3 or **B** iBCEC cells or **C** primary BCEC with or without 4 h EGTA preincubation (5 mM). Data represents at least seven replicates from three independent experiments, mean ± SEM. Simple linear regression was performed and the resulting slopes used to calculate the permeability coefficient P_app_ (Table 1). Statistical significance was tested for all cell types with two-way ANOVA and significances between groups at each time point are depicted in each graph (from top to bottom: blue = empty wells vs. EGTA; black = untreated cells vs. EGTA; grey = empty well vs. cells). The in-detail list of statistical results is shown in Table S6. **D** Clearance volume for each cell type was normalized to the respective filter for comparison of cell layer influence on permeability. Minimal slope of linear regression indicates no relevant time dependence of the normalized clearance volume. There was a significant difference between all cell models (*p* < 0.0001), except iBCEC and primary BCEC. This statistical analysis was performed with two-way ANOVA with Turkey’s multiple comparison test by comparing the column means

ᴅ-Tyr(Me)Orn was then used to evaluate the permeability of monolayers of three different endothelial cell models: The immortalized cell line hCMEC/D3, primary BCECs and iPS derived BCEC (Fig. 4). The permeability coefficients P_app_ was calculated for each cell type and is shown in Table 1. While hCMEC/D3 monolayers did not show a large effect on the permeability of ᴅ-Tyr(Me)Orn through the Transwell^®^ (Fig. 4, A), which is also reflected in the high P_app_ values (Table 1), primary and iBCEC restricted its flow stronger (Fig. 4, B and C). This effect could be partly reversed by EGTA. The impact of all cell monolayers is shown in Fig. 4D. To compare all used cell types in Transwells^®^, the clearance volumes of untreated cells was normalized to the respective empty filter control, and is given as the % of the respective empty filter.

**Table 1:** Apparent permeability coefficients of ᴅ-Tyr(Me)Orn

		Empty wells	5 mM EGTA	Untreated
MDCK II	P_app_ [cm/s]	4.08 ± 0.30 × 10^− 5^	3.09 ± 0.36 × 10^− 5^	5.06 ± 1.06 × 10^− 7^
	corr. P_app_ [cm/s]		1.35 ± 0.35 × 10^− 4^	5.13 ± 1.08 × 10^− 7^
hCMEC/D3	P_app_ [cm/s]	3.41 ± 0.90 × 10^− 5^	2.85 ± 0.49 × 10^− 5^	2.93 ± 0.42 × 10^− 5^
	corr. P_app_ [cm/s]		9.14 ± 4.58 × 10^− 5^	7.63 ± 3.82 × 10^− 5^
iBCEC	P_app_ [cm/s]	1.76 ± 0.18 × 10^− 4^	1.06 ± 0.16 × 10^− 4^	7.51 ± 2.22 × 10^− 5^
	corr. P_app_ [cm/s]		9.50 ± 9.29 × 10^− 3^	1.46 ± 0.54 × 10^− 4^
primary BCEC	P_app_ [cm/s]	1.56 ± 0.05 × 10^− 4^	1.10 ± 0.15 × 10^− 4^	6.92 ± 1.79 × 10^− 5^
	corr. P_app_ [cm/s]		4.79 ± 1.95 × 10^− 4^	1.53 ± 0.72 × 10^− 4^

P_app_ refers to the total permeability coefficient, while corrected P_app_ of treated and untreated cells were calculated by subtracting the respective filter effect, as described in the method section. Mean ± SEM was calculated from three independent experiments

### Paracellular marker performance in comparison to standard fluorescent permeability markers Lucifer Yellow and fluorescein

**Fig. 5:**
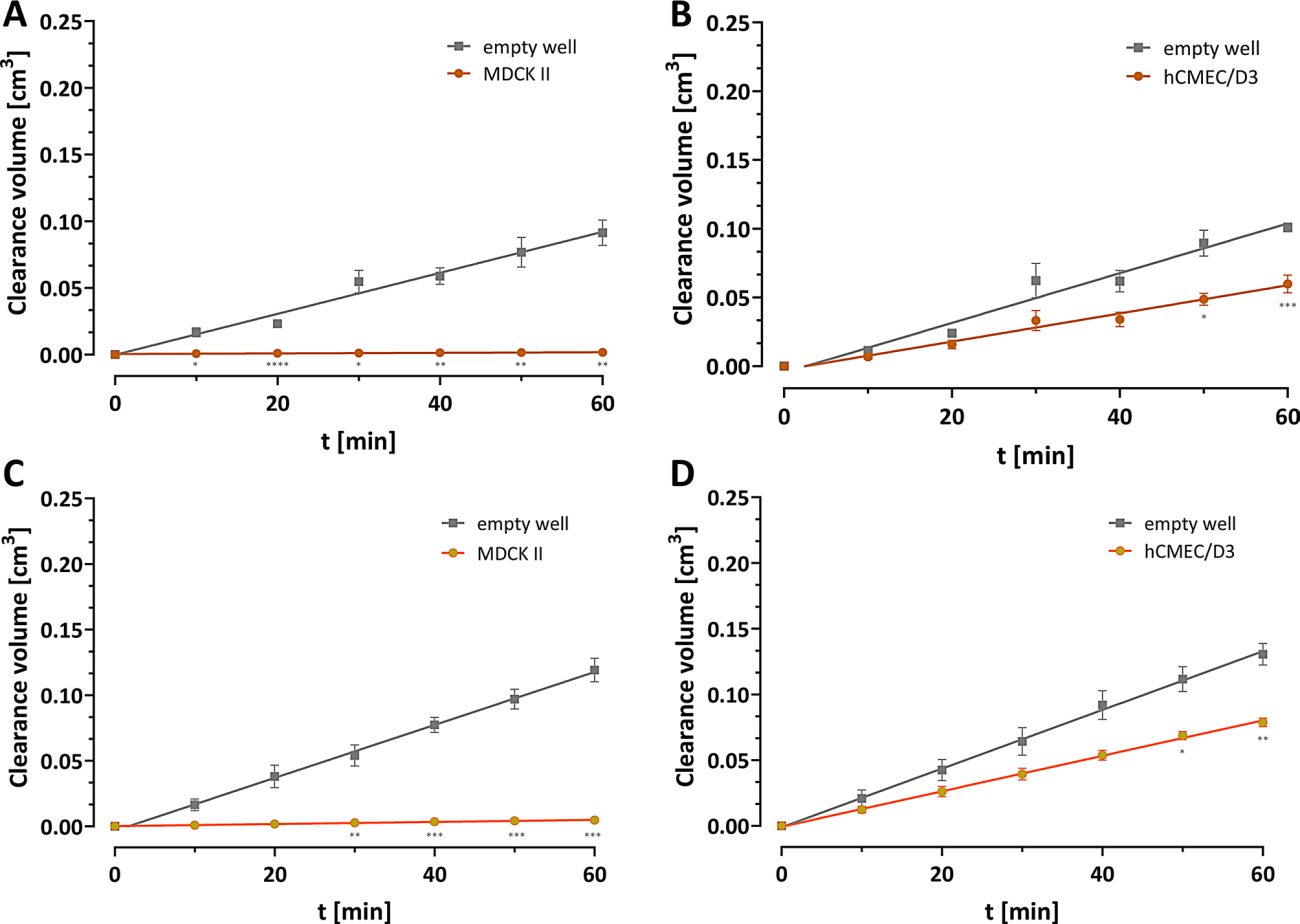
Permeability of **A and B** Lucifer Yellow and **C and D** fluorescein, shown as the clearance volume over time from the apical to the basolateral compartment of Transwells^®^ either in empty wells or in wells seeded with either confluent **A and C** MDCK II cells or **B and D** hCMEC/D3 cells. Data represents at least six replicates from three independent experiments (mean ± SEM). Simple linear regression was performed and the resulting slopes were used to calculate the permeability coefficient P_app_ (see Table 2). Statistical significance was tested for all cell types using a mixed-effects analysis and significances between the two groups at each time point are depicted in each graph. The in-detail list of the statistical results is shown in Table S7

To compare the performance of ᴅ-Tyr(Me)Orn as a paracellular marker and to validate the results externally, we performed Transwell^®^ experiments using the standard fluorescent marker Lucifer Yellow and fluorescein, in the cell models with the highest difference, MDCK II and hCMEC/D3. As can be seen in Fig. 5A and B, the Lucifer Yellow results were comparable with those of ᴅ-Tyr(Me)Orn resulting in similar P_app_ values (see Table 2). The qualitative results for fluorescein were the same, while the difference between the cell models was reduced to one order of magnitude.

**Table 2:** Apparent permeability coefficients of Lucifer Yellow and fluorescein

		Lucifer Yellow		Fluorescein	
		Empty wells	Untreated	Empty wells	Untreated
MDCK II	P_app_ [cm/s]	2.29 ± 0.05 × 10^− 5^	3.28 ± 1.20 × 10^− 7^	3.00 ± 0.01 × 10^− 5^	1.18 ± 0.75 × 10^− 6^
	corr. P_app_ [cm/s]		3.29 ± 1.20 × 10^− 7^		1.18 ± 0.75 × 10^− 6^
hCMEC/D3	P_app_ [cm/s]	2.69 ± 0.07 × 10^− 5^	1.53 ± 0.28 × 10^− 5^	3.32 ± 0.09 × 10^− 5^	2.01 ± 0.06 × 10^− 5^
	corr. P_app_ [cm/s]		4.31 ± 1.8 × 10^− 5^		5.10 ± 0.52 × 10^− 5^

P_app_ referrers to the total permeability coefficient, while corrected P_app_ of treated and untreated cells were calculated by subtracting the respective filter effect, as described in the method section. Mean ± SEM was calculated from three independent experiments

### Distribution, pharmacokinetics and brain-to-blood ratio of ᴅ-Tyr(Me)Orn in mice

**Fig. 6:**
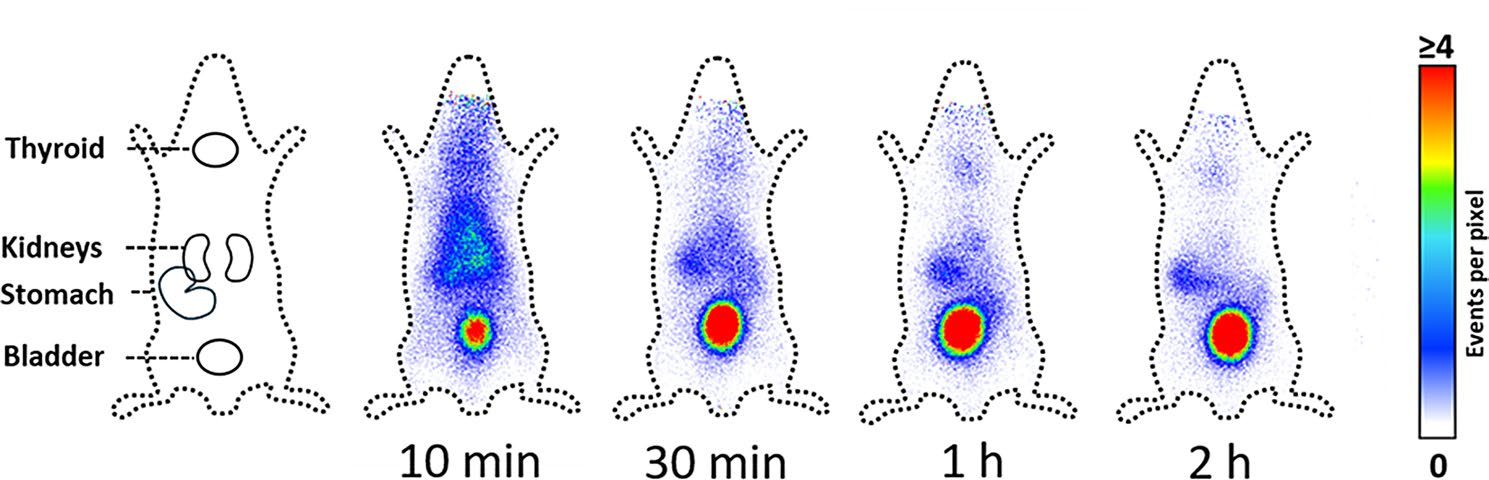
Distribution of ᴅ-Tyr(Me)Orn labeled with ^125^I and injected in healthy mice. Pixel intensity scale is given on the right

The in vivo distribution of the novel marker was evaluated after *i.v.* injection with radiolabeled (^125^I) ᴅ-Tyr(Me)Orn over the course of 2 h in healthy mice (Fig. 6). An apparent exclusive distribution in the systemic circulation was observed with a rapid renal clearance. Residual radioactivity accumulation in thyroid and stomach is consistent with free ^125^I accumulation and presumably caused by (enzymatic) release of ^125^I from the dipeptide [28].

We then evaluated the free fraction of ᴅ-Tyr(Me)Orn in mouse plasma. Overall, the protein binding was low. For the three tested concentration, 10 ng/mL resulted in 94.7 ± 3.1%; 100 ng/ml in 97.3 ± 1.8%; and 2000 ng/mL in 89.9 ± 3.4% fraction unbound.

**Fig. 7:**
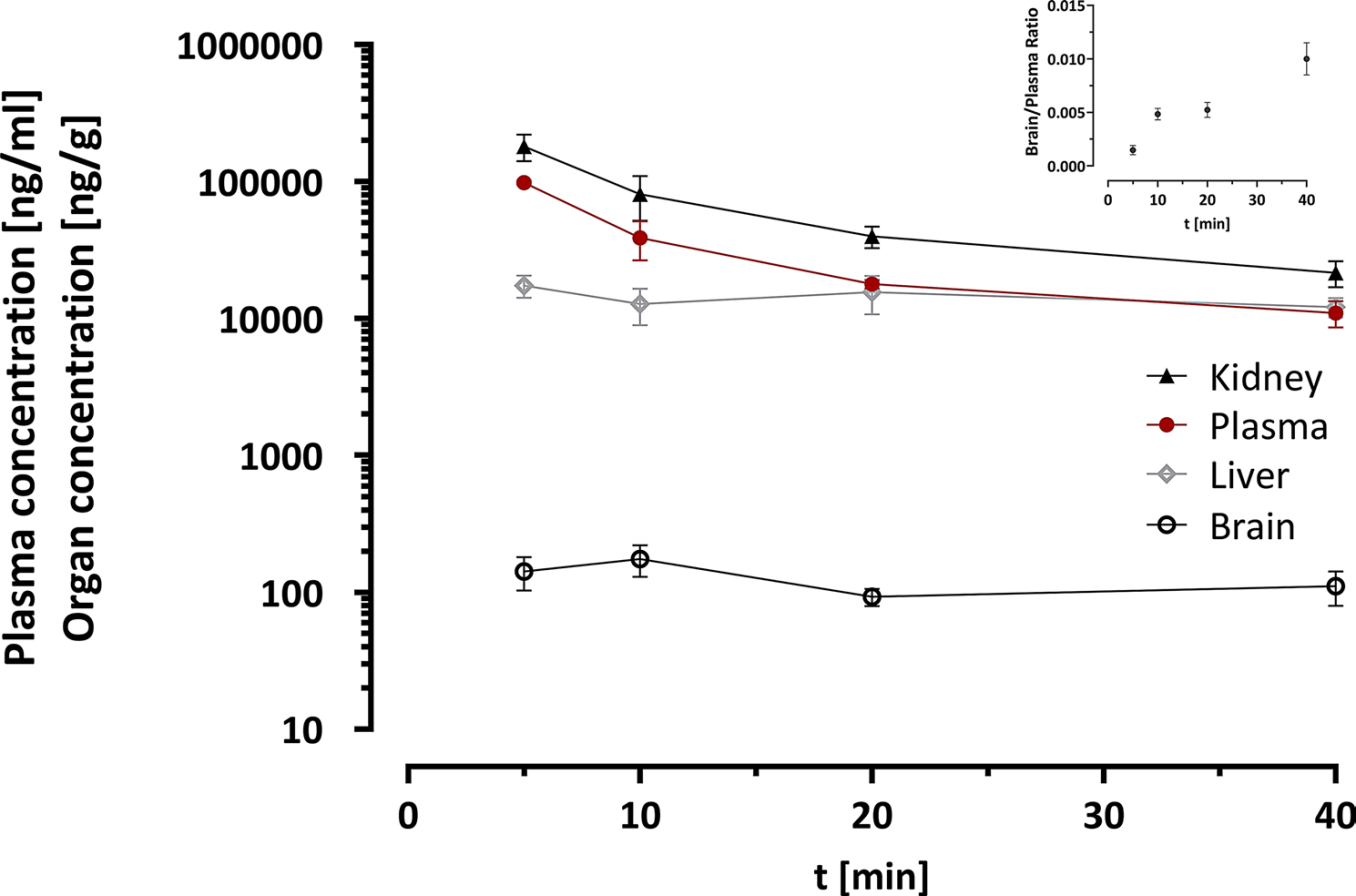
ᴅ-Tyr(Me)Orn concentration time profile in liver, kidney, brain and plasma after *i.v.* injection and PBS perfusion in healthy mice. Inlayed graph on the right upper corner depicts the brain-to-plasma ratio at the observed time points. Data is represented as the mean ± SEM from three mice at each time point

Subsequently, the concentration of ᴅ-Tyr(Me)Orn in plasma, brain, liver, and kidney 5, 10, 20 and 40 min after *i.v.* injection was measured with UPLC-MS/MS (Fig. 7). The pharmacokinetic analysis of plasma concentration time profile revealed an AUC_∞_ of 2129.5 min*µg/mL with a half-life (t_1/2_) of 28.7 min and a clearance CL of 0.48 ml/min. The theoretical C_0_ was 248 µg/mL. The extrapolated steady state distribution volume V_ss_ was 11.89 mL.

Consistent with the renal clearance observed through imaging, the kidneys showed a parallel profile to the plasma concentration with a slightly higher C_0_ of 404 µg/g and an AUC_∞_ of 3874.9 min*µg/g, with t_1/2_ of 22.6 min. The liver concentration stayed constant over the whole observed period and was comparable to plasma at 20 and 40 min. Only very low amounts of ᴅ-Tyr(Me)Orn reached the brain (142 ng/g after 5 min), equaling 0.0064 ± 0.003% of injected dose, and this concentration stayed constant during the observed time frame. Compared to the concentration in plasma, the mean brain-to-plasma ratio increased from 0.0015 to 0.01 from 5 to 40 min, based on the decreasing plasma concentration.

## Discussion

The permeability across physiological barriers is one of the most important parameters in drug development and relevant preclinical in vitro screenings, as well as in gaining mechanistic insights into the modulation of barrier integrity. Especially the BBB stringently regulates access of substances to the brain and, as a consequence, provides a major challenge for the effective treatment of CNS disorders. It is crucial to evaluate the BBB permeability during drug development to assure an effective therapy behind the BBB and to avoid CNS side effects for drugs with peripheral targets. To acquire this knowledge, preclinical models must be reliable. Achieving strong BBB restriction has been a key focus of in vitro model development, ensuring translational relevance. Using paracellular markers to assess paracellular flux is a viable way to test the integrity of tight junctions and to investigate any effects on them. It also provides an evaluation of individual replicates (e.g. Transwell^®^) of such models, enhancing data interpretation and robustness. This is especially valuable as an internal control in permeation experiments of drug candidates, in investigation of pathological mechanisms and of tight junction modulators for drug delivery. In this context, permeability markers can help to validate the used models and simultaneously normalize the generated permeability coefficients, especially if they have a comparable size to tested drugs. At each measured time point, the clearance volume can be normalized to the amount of cleared paracellular marker substance, and therefore correct for any impairment of the cell monolayers (for example by damaging with pipettes or effects of other experimental parameters). Differentiating between trans- and paracellular mechanisms is essential for accurate conclusions on drug candidate designs or effects in pathological conditions. However, currently used paracellular markers either lack relevance, sensitivity, require large sample volume or use radioactivity. While saccharides, when used as a permeability marker, are normally applied at concentrations (10 mg/kg [17]) lower than when used as hyperosmolar solution for BBB opening (20–25% solution [5, 7]) – they do carry the risk of BBB impairment by osmotic pressure and therefore inaccurate results.

### ᴅ-Tyr(Me)Orn can be sensitively quantified by UPLC-MS/MS

The drawbacks of established permeability markers prompted us to develop and validate a new paracellular marker that does not suffer from these weaknesses. In particular, our novel paracellular marker can be highly sensitively quantified with UPLC-MS/MS, thereby providing increased reliability for low permeation. The pertinent quantification assay fulfills all applicable recommendations of the guideline for bioanalytical method validation [25], which demonstrates its reliability and robustness. The assay was validated in four different matrices: cell culture medium for Transwell^®^ experiments, cell lysates for intracellular quantifications, mouse plasma and mouse brain proving its applicability for all stages of drug development from discovery to in vivo pharmacokinetics and biodistribution experiments. Its low limit of quantification combined with its small required sample volume allows its use in various experimental settings and the possibility to use samples for simultaneous measurements of other compounds like drug candidates. ᴅ-Tyr(Me)Orn can be used as an internal control of tight junction status during permeation experiments enabling more accurate conclusions on permeation mechanisms or improving data analysis by identifying compromised samples. The withdrawn volume in our experiments is significantly lower than in conventional Transwell^®^ experiments. While it is considered small enough to reduces interferences during sampling and large enough to allow remeasurement and minimize pipetting errors, the concentration is still high enough to allow further reduction of the volume if warranted. UPLC-MS/MS measurements allow to parallelly quantify drugs label-free. While the hydrophilicity of the marker required a hydrophilic interaction chromatography in our hands, the extracts can easily be split to allow quantification from aqueous samples after solvent evaporation or dilution to suite reverse-phase LC-MS quantification, the primary technique for usually more lipophilic drugs.

### ᴅ-Tyr(Me)Orn is a valid marker of paracellular flux

The novel dipeptide marker ᴅ-Tyr(Me)Orn consisting of the non-canonical ᴅ-amino acids ᴅ-O-methyl-tyrosine and ᴅ-ornithine is a feasible alternative to existing paracellular markers due to its small size and hydrophilicity. Additionally, it can readily and easily be synthesized from commercially available reagents. Despite being a dipeptide, its composition of non-canonical ᴅ-amino acids should result in biological inertness: it should not be recognized by hydrolyzing enzymes, solute carriers, and transporters. While the crossing of dipeptides over the BBB is in itself unlikely, because only specific ones have been shown to be transported [29], the exclusive use of ᴅ-amino acids abolishes the substrate characteristics for both PepT1 and PepT2 [30–32]. Aside from that, a relevant expression of PepT’s uptake on the BBB is debated [29, 33–35] and we demonstrated that ᴅ-Tyr(Me)Orn is not recognized by PepT1. This also rules out a possible binding by PepT2, given the significant overlap in substrate specificities of PepT1 and PepT2 [36, 37]. In contrast to PepTs, LAT-1 is highly expressed on the BBB and facilitates the import of amino acids into the brain [38, 39]. Moreover, computational modelling has proposed a potential recognition of dipeptides [40]. However, our data does prove that ᴅ-Tyr(Me)Orn is also no substrate of LAT-1. Taken together, we obtained no evidence that ᴅ-Tyr(Me)Orn is actively transported verifying its feasibility as a marker for paracellular flux.

We substantiated this by confirming that negligible uptake of ᴅ-Tyr(Me)Orn into hCMEC/D3 cells takes place, reflected by the low remaining amount in the well, even lower than other paracellular markers which is orders of magnitude lower than of actively transported substances like GlySar or leucine. The hCMEC/D3 cell line is a well-known and well-studied BBB model. Although the tightness of this model is not accurately reproducing the physiological BBB, the expression profile of transporters equals primary capillary endothelial cells [41–43], and it is therefore considered a suitable model for intracellular uptake in BCEC.

After we ruled out that ᴅ-Tyr(Me)Orn can be taken up into BCECs, we confirmed the paracellular flux marker characteristics by assessing the P_app_ in the Transwell^®^ system with a gold-standard cell-model for permeability investigations, MDCK II, known to form very tight monolayers [44, 45]. Evaluation of the MDCK II permeability demonstrated the required low P_app_ of a true paracellular marker representative of the restrictive properties of physiologically relevant barriers, with 2 orders of magnitude difference between the empty filter and the cell monolayer. The significant impact of opening the adherens junctions by calcium complexation via EGTA treatment further demonstrated the paracellular flux assessment feasibility of ᴅ-Tyr(Me)Orn, because it can discriminate between intact and weakened junctions. As an additional orthogonal validation, we performed Transwell^®^ experiments with two standard permeability marker substances: Lucifer Yellow and fluorescein. Both fluorophores produced P_app_ values that were consistent with previous publications [46, 47]. Lucifer Yellow performed similarly to the paracellular marker ᴅ-Tyr(Me)Orn, while fluorescein exhibited a reduced discriminatory power by one order of magnitude. These results, together with the absence of active transport mechanisms and uptake into hCMEC/D3 cells validates ᴅ-Tyr(Me)Orn as a reliable marker for paracellular flux.

### Readily available BBB cell monolayer models suffer from low restrictive characteristics

After confirming ᴅ-Tyr(Me)Orn as a marker for paracellular tightness, we compared available monolayer models of the BBB and tested three BCEC cell types in the Transwell^®^ system. The already mentioned hCMEC/D3 model does not form tight barriers, which was confirmed with our ᴅ-dipeptide, whose flux into the basolateral compartment was almost not hindered by the cell monolayer. In contrast, primary BCECs [48] and iBCECs are reported to form tighter barriers with TEER values for iBCEC reaching the range of proposed in vivo values [49, 50]. As expected, our iBCEC and primary BCEC demonstrated superior tight junction functionality over hCMEC/D3 in the context of BBB models. However, we observed that they achieve a substantially weaker barrier than MDCK II cells, as demonstrated with ᴅ-Tyr(Me)Orn permeability values. Therefore, one can conclude that the brain capillary endothelial cell models as herein used, are not exhibiting relevant BBB tight junction integrity in the context of mono-culture. There is rapid progress for novel methodologies for iBCEC differentiation in the field, additionally discussing the epithelial vs. endothelial phenotype of these cells [51]. Evaluation of generated iBCEC with the reliable permeability marker ᴅ-Tyr(Me)Orn, could provide robust and comparable insights for different differentiation protocols regarding the tightness of these barriers. Also, in all models, EGTA was used to verify that the reduction of the P_app_ by the cell monolayer is due to cell-cell-connections, further confirming the feasibility of our ᴅ-dipeptide as a paracellular flux marker. The observed large permeability differences for the different cell monolayers in the Transwells^®^ demonstrates the capacity of the ᴅ-dipeptide to evaluate the paracellular tightness of BBB models.

### ᴅ-Tyr(Me)Orn shows low brain penetration in mice

Lastly, we tested the applicability of our marker in vivo, which resulted in 0.006% of injected dose in the brain between 5 and 40 min after injection and very low brain-to-blood ratios during the observed time frame. The radioactive imaging via (^125^I)-labeling showed a rapid renal elimination, which is expected for a small hydrophilic molecule. This was confirmed with the preliminary pharmacokinetic profile from 5 to 40 min, which gives important information for the future in vivo use. The half-life was determined to be 20–30 min and the protein binding analysis revealed that the largest part of ᴅ-Tyr(Me)Orn is unbound. The volume of distribution of 0.354 ml/g indicates low tissue distribution.

We observed constant liver concentrations that were comparable to plasma concentrations between 20 and 40 min. As expected for this highly vascularized organ with fenestrated capillary vessels, the liver concentrations were significantly higher than those in the brain.

The lower concentration in the brain stayed also constant. The resulting estimated brain-to-plasma ratio would be 0.006%, but the brain-to-plasma ratio of 0.15% at the first sampling after 5 min might be more representative. For comparison with our in vitro experiments: 0.14% of uptake solution reached the lower compartment of the Transwell^®^ through the MDCK II monolayer after 60 min and ~ 7% for hCMEC/D3. In summary, this data confirms the applicability of ᴅ-Tyr(Me)Orn as a BBB integrity marker in vivo.

This study has limitations: (1) While we used well-known cell lines in the Transwell^®^ system and verified them with the well-known permeability markers Lucifer Yellow and fluorescein, we did not compare the permeability of ᴅ-Tyr(Orn) with TEER measurements, because the latter require experienced handling and are prone to error. Nevertheless, we believe that our data confirms the potential of ᴅ-Tyr(Me)Orn to differentiate between the tightness of barriers.

(2) Although, to the best of our knowledge, we excluded biological activity by rational design and excluded most probable routes of transcellular uptake in vitro in cell lines, we cannot completely exclude that there is a so far undetected possible transcellular route for ᴅ-Tyr(Me)Orn in primary cells or in vivo. However, our data does not indicate that such transport could play a relevant role, if present at all.

(3) In our in vivo experiments, we only assessed the pharmacokinetics and BBB permeability in mice with intact BBB. The evaluation of ᴅ-Tyr(Me)Orn as in vivo marker for BBB impairment in mice with disrupted BBB should be performed in future investigations. However, we believe that our results underline the potential of this novel d-dipeptide marker for in vivo BBB permeability experiments.

## Conclusion

In this study we designed and evaluated a novel BBB paracellular permeability marker consisting of the non-canonical ᴅ-amino acids ᴅ-O-methyl-tyrosine and ᴅ-ornithine, and established its highly sensitive UPLC-MS/MS assay in different biological matrices. We validated the feasibility of ᴅ-Tyr(Me)Orn as paracellular flux marker and demonstrated its use in the context of BBB permeability studies, including in vitro model evaluation and in vivo brain uptake experiments. Therefore, it could be viable for investigations of BBB modulation in pathological conditions like stroke [4] or sepsis [52]. Used as the lower threshold for brain permeability, it can be used for in vivo and in vitro normalization for pharmacological evaluations of drug candidates BBB permeability. This can increase the comparability of results and can help to identify and exclude artefacts due to impaired barrier function. Evaluations of BBB models with a harmonized and easily sensitively measured marker could provide a powerful tool for standardization in this research field. The commonly used markers cannot be measured with comparable sensitivity and therefore require high concentrations or necessitate radioactive labelling, can be toxic, or might open the BBB through osmotic pressure. In contrast, the highly sensitive, label-free measurement of our new permeability marker allows for low doses/concentrations and therefore shows an advantage over saccharides in general. Because of its all-d configuration the marker is resistant against proteolytic digestion and shows absence of solute carrier and transporter substrate characteristics. It can therefore be used as paracellular marker not only at the BBB but also at different barriers, for example across the gastro-intestinal mucosa after oral administration.

## Supplementary Information

Below is the link to the electronic supplementary material.

Supplementary Material 1: Table S1 parameters for the MS/MS detection of ᴅ-Tyr(Me)Orn and ᴅ-Tyr([2H3]Me)Orn in positive heated ESI and SRM. Table S2: Validation results for the ᴅ-Tyr(Me)Orn assay in the four different biological matrices. Table S3: IS normalized matrix effect and recovery. Figure S4: Representative chromatograms of study samples. Figure S5: Remaining A Lucifer Yellow and B fluorescein percentage of uptake solution (100 and 300 µM) in 96-well plates. Table S6: Statistical analysis of ᴅ-Tyr(Me)Orn Transwell^®^ assays. Table S7: Statistical analysis of Lucifer Yellow and fluorescein Transwell^®^ assays.

## Data Availability

The data supporting the conclusions of this article is included within the published article. ᴅ-Tyr(Me)Orn generated in this study is available from the corresponding author upon request.

## Abbreviations

BBB: Blood brain-barrier
UPLC-MS/MS: Ultra-performance liquid chromatography tandem mass spectrometry
ᴅ-Tyr(Me)Orn: ᴅ-O-methyl-tyrosine and ᴅ-ornithine
CNS: Central nervous system
TJ: Tight junctions
TEER: Transendothelial resistance
FITC: Fluorescein isothiocyanate
MW: Molecular weight
LLOQ: Lower limit of quantification
iPS: Induced pluripotent stem cells
BCEC: Brain capillary endothelial cells
DMEM: Dulbecco’s Modified Eagle’s Medium
NEAS: Non-essential amino acid solution
HBSS: Hanks Balanced Salt Solution
GlySar: Glycil-sarcosine
FBS: Fetal bovine serum
PBS: Phosphate-buffered saline
EtOAc: Ethyl acetat
EtOH: Ethanol
MeOH: Methanol
DCM: Dichloromethane
RPMI: Roswell Park Memorial Institute
EGTA: Ethylene glycol tetraacetic acid
Fmoc-OSu: Fmoc N-hydroxysuccinimide ester
HBTU: 2-(1 H-benzotriazol-1-yl)-1,1,3,3-tetramethyluronium hexafluorophosphate
DIEA: Diisopropylethylamine
TFA: Trifluoroacetic acid
DMF: Dimethylformamide
ACN: Acetonitrile
FA: Formic acid
EGM: Endothelial Growth Medium 2 with all supplements of the EGM 2 kit
iBCEC: IPS-derived BCEC
Medium-HS: Medium with human serum
HHBSS: HBSS with 0.01 M Hepes
SRM: Selected reaction monitoring
CID: Collision-induced dissociation
ESI: Electrospray ionization
QC: Quality control

## References

[1] Greene C, Hanley N, Campbell M. Claudin-5: gatekeeper of neurological function. Fluids barriers CNS. 2019;16:3. 30691500 10.1186/s12987-019-0123-z PMC6350359

[2] Lochhead JJ, Yang J, Ronaldson PT, Davis TP. Structure, function, and regulation of the blood-brain barrier tight junction in central nervous system disorders. Front Physiol. 2020;11:914. 32848858 10.3389/fphys.2020.00914 PMC7424030

[3] Montagne A, Zhao Z, Zlokovic BV. Alzheimer’s disease: a matter of blood-brain barrier dysfunction? J Exp Med. 2017;214:3151–69. 29061693 10.1084/jem.20171406 PMC5679168

[4] Liu J, Jin X, Liu KJ, Liu W. Matrix metalloproteinase-2-mediated occludin degradation and caveolin-1-mediated claudin-5 redistribution contribute to blood-brain barrier damage in early ischemic stroke stage. J Neurosci. 2012;32:3044–57. 22378877 10.1523/JNEUROSCI.6409-11.2012 PMC3339570

[5] Doolittle ND, Miner ME, Hall WA, Siegal T, Hanson EJ, Osztie E, et al. Safety and efficacy of a multicenter study using intraarterial chemotherapy in conjunction with osmotic opening of the blood-brain barrier for the treatment of patients with malignant brain tumors. Cancer. 2000;88:637–47. 10649259 10.1002/(sici)1097-0142(20000201)88:3<637::aid-cncr22>3.0.co;2-y

[6] McDannold N, Arvanitis CD, Vykhodtseva N, Livingstone MS. Temporary disruption of the blood-brain barrier by use of ultrasound and microbubbles: safety and efficacy evaluation in rhesus macaques. Cancer Res. 2012;72:3652–63. 22552291 10.1158/0008-5472.CAN-12-0128 PMC3533365

[7] Sato S, Kawase T, Harada S, Takayama H, Suga S. Effect of hyperosmotic solutions on human brain tumour vasculature. Acta Neurochir. 1998;140:1135–41. 9870058 10.1007/s007010050227

[8] Inoue S, Shirakura K, Shirono A, Taguchi J, Ikeda Y, Tomita S, et al. Claudin 5-binding small molecule transiently opens the blood-brain barrier and safely enhances brain drug delivery. J Control Release. 2025;388:114314. 41083006 10.1016/j.jconrel.2025.114314

[9] Di Marco A, Gonzalez Paz O, Fini I, Vignone D, Cellucci A, Battista MR, et al. Application of an in vitro blood-brain barrier model in the selection of experimental drug candidates for the treatment of Huntington’s Disease. Mol Pharm. 2019;16:2069–82. 30916978 10.1021/acs.molpharmaceut.9b00042

[10] Goldmann EE. Vitalfärbung am Zentralnervensystem: Beitrag zur Physio-Pathologie des Plexus Chorioideus und der Hirnhäute. Verlag der königl. Akademie der Wissenschaften; 1913.

[11] Ehrlich P. Das Sauerstoff- Bedürfniss des Organismus: Eine farbenanalytische Studie. Verlag von August Hirschwald, Berlin. 1885.

[12] Saunders NR, Dziegielewska KM, Mollgard K, Habgood MD. Markers for blood-brain barrier integrity: how appropriate is Evans blue in the twenty-first century and what are the alternatives? Front Neurosci. 2015;9:385. 26578854 10.3389/fnins.2015.00385 PMC4624851

[13] Dragojevic J, Marakovic N, Popovic M, Smital T. Zebrafish (Danio rerio) Oatp2b1 as a functional ortholog of the human OATP2B1 transporter. Fish Physiol Biochem. 2021;47:1837–49. 34546486 10.1007/s10695-021-01015-7

[14] Choi JJ, Wang S, Tung YS, Morrison B 3rd, Konofagou EE. Molecules of various pharmacologically-relevant sizes can cross the ultrasound-induced blood-brain barrier opening in vivo. Ultrasound Med Biol. 2010;36:58–67. 10.1016/j.ultrasmedbio.2009.08.006 PMC2997717 19900750

[15] Nitta T, Hata M, Gotoh S, Seo Y, Sasaki H, Hashimoto N, et al. Size-selective loosening of the blood-brain barrier in claudin-5-deficient mice. J Cell Biol. 2003;161:653–60. 12743111 10.1083/jcb.200302070 PMC2172943

[16] Brooks TA, Ocheltree SM, Seelbach MJ, Charles RA, Nametz N, Egleton RD, et al. Biphasic cytoarchitecture and functional changes in the BBB induced by chronic inflammatory pain. Brain Res. 2006;1120:172–82. 17007822 10.1016/j.brainres.2006.08.085 PMC3893032

[17] Noorani B, Chowdhury EA, Alqahtani F, Ahn Y, Patel D, Al-Ahmad A, et al. LC-MS/MS-based in vitro and in vivo investigation of blood-brain barrier integrity by simultaneous quantitation of mannitol and sucrose. Fluids barriers CNS. 2020;17:61. 33054801 10.1186/s12987-020-00224-1 PMC7556948

[18] Franke H, Galla H-J, Beuckmann CT. An improved low-permeability in vitro-model of the blood–brain barrier: transport studies on retinoids, sucrose, haloperidol, caffeine and mannitol. Brain Res. 1999;818:65–71. 9914438 10.1016/s0006-8993(98)01282-7

[19] Patabendige A, Skinner RA, Morgan L, Abbott NJ. A detailed method for preparation of a functional and flexible blood-brain barrier model using porcine brain endothelial cells. Brain Res. 2013;1521:16–30. 23603406 10.1016/j.brainres.2013.04.006 PMC3694295

[20] Licea-Perez H, Junnotula V, Zohrabian S, Karlinsey M. Development of a multi-sugar LC-MS/MS assay using simple chemical derivatization with acetic anhydride. Anal Methods. 2016;8:3023–33.

[21] Miah MK, Bickel U, Mehvar R. Development and validation of a sensitive UPLC-MS/MS method for the quantitation of [(13)C]sucrose in rat plasma, blood, and brain: Its application to the measurement of blood-brain barrier permeability. J Chromatogr B. 2016;1015–1016:105–10. 10.1016/j.jchromb.2016.02.017 26919445

[22] von Linde T, Bajraktari-Sylejmani G, Haefeli WE, Burhenne J, Weiss J, Sauter M. Rapid and sensitive quantification of intracellular glycyl-sarcosine for semi-high-throughput screening for inhibitors of PEPT-1. Pharmaceutics. 2021;13:1019. 34371711 10.3390/pharmaceutics13071019 PMC8309108

[23] Crim JW, Garczynski SF, Brown MR. Approaches to radioiodination of insect neuropeptides. Peptides. 2002;23:2045–51. 12431743 10.1016/s0196-9781(02)00192-4

[24] Bay C, Bajraktari-Sylejmani G, Haefeli WE, Burhenne J, Weiss J, Sauter M. Functional characterization of the solute carrier LAT-1 (SLC7A5/SLC2A3) in human brain capillary endothelial cells with rapid UPLC-MS/MS quantification of intracellular isotopically labelled L-leucine. Int J Mol Sci. 2022;23:5029. 35408997 10.3390/ijms23073637 PMC8998838

[25] International Council for Harmonisation of Technical Requirements for Pharmaceuticals for Human Use. Bioanalytical method validation and sample analysis M10. 2022. https://database.ich.org/sites/default/files/M10_Guideline_Step4_2022_0524.pdf. Accessed 14 Nov 2025.

[26] Matuszewski BK. Standard line slopes as a measure of a relative matrix effect in quantitative HPLC-MS bioanalysis. J Chromatogr B. 2006;830:293–300. 10.1016/j.jchromb.2005.11.009 16310419

[27] Pardridge WM, Triguero D, Cancilla JY. Comparison of in vitro and in vivo models of drug transcytosis through the blood-brain barrier. J Pharmacol Exp Ther. 1990;253:884–91. 2338660

[28] Cavina L, van der Born D, Klaren PHM, Feiters MC, Boerman OC, Rutjes F. Design of radioiodinated pharmaceuticals: structural features affecting metabolic stability towards in vivo deiodination. Eur J Org Chem. 2017;2017:3387–414. 10.1002/ejoc.201601638 PMC5499721 28736501

[29] Tanaka M, Dohgu S, Komabayashi G, Kiyohara H, Takata F, Kataoka Y, et al. Brain-transportable dipeptides across the blood-brain barrier in mice. Sci Rep. 2019;9:5769. 30962462 10.1038/s41598-019-42099-9 PMC6453885

[30] Meredith D, Boyd CAR. Oligopeptide transport by epithelial cells. J Membrane Biol. 1995;145:1–12. 7636881 10.1007/BF00233302

[31] Boyd CAR, Ward MR. A micro-electrode study of oligopeptide absorption by the small intestinal epithelium of necturus maculosus. J Physiol. 1982;324:411–28. 7097606 10.1113/jphysiol.1982.sp014121 PMC1250714

[32] Lister N, Sykes AP, Bailey PD, Boyd CAR, Bronk JR. Dipeptide transport and hydrolysis in isolated loops of rat small intestine: effects of stereospecificity. J Physiol. 1995;484:173–82. 7602518 10.1113/jphysiol.1995.sp020656 PMC1157930

[33] Parvez MM, Sadighi A, Ahn Y, Keller SF, Enoru JO. Uptake transporters at the blood-brain barrier and their role in brain drug disposition. Pharmaceutics. 2023;15:2473. 37896233 10.3390/pharmaceutics15102473 PMC10610385

[34] Smith DE, Clemencon B, Hediger MA. Proton-coupled oligopeptide transporter family SLC15: physiological, pharmacological and pathological implications. Mol Aspects Med. 2013;34:323–36. 23506874 10.1016/j.mam.2012.11.003 PMC3602806

[35] Hu Y, Xie Y, Keep RF, Smith DE. Divergent developmental expression and function of the proton-coupled oligopeptide transporters PepT2 and PhT1 in regional brain slices of mouse and rat. J Neurochem. 2014;129:955–65. 24548120 10.1111/jnc.12687 PMC4181614

[36] Fei Y-J, Liu J-C, Fujita T, Liang R, Ganapathy V, Leibach FH. Identification of a potential substrate binding domain in the mammalian peptide transporters PEPT1 and PEPT2 using PEPT1-PEPT2 and PEPT2-PEPT1 chimeras. Biochem Biophys Res Commun. 1998;246:39–44. 9600064 10.1006/bbrc.1998.8566

[37] Killer M, Jiri Wald J, Pieprzyk J, Marlovits TC, Löw C. Structural snapshots of human PepT1 and PepT2 reveal mechanistic insights into substrate and drug transport across epithelial membranes. Sci Adv. 2021;7:eabk3259. 34730990 10.1126/sciadv.abk3259 PMC8565842

[38] Kanai Y, Segawa H, Miyamoto K, Uchino H, Takeda E, Endou H. Expression cloning and characterization of a transporter for large neutral amino acids activated by the heavy chain of 4F2 antigen (CD98). J Biol Chem. 1998;273:23629–32. 9726963 10.1074/jbc.273.37.23629

[39] Boado RJ, Li JY, Nagaya M, Zhang C, Pardridge WM. Selective expression of the large neutral amino acid transporter at the blood–brain barrier. Proc Natl Acad Sci U S A. 1999;96:12079–84. 10518579 10.1073/pnas.96.21.12079 PMC18415

[40] Khavinson VK, Linkova NS, Rudskoy AI, Petukhov MG. Feasibility of transport of 26 biologically active ultrashort peptides via LAT and PEPT family transporters. Biomolecules. 2023;13:552. 36979488 10.3390/biom13030552 PMC10046148

[41] Weksler BB, Subileau EA, Perrière N, Charneau P, Holloway K, Leveque M, et al. Blood-brain barrier-specific properties of a human adult brain endothelial cell line. FASEB J. 2005;1872–4. 10.1096/fj.04-3458fje 16141364

[42] Taggi V, Schafer AM, Dolce A, Meyer Zu Schwabedissen HE. A face-to-face comparison of the BBB cell models hCMEC/D3 and hBMEC for their applicability to adenoviral expression of transporters. J Neurochem. 2024;168:2611–20. 38735840 10.1111/jnc.16125

[43] Ohtsuki S, Ikeda C, Uchida Y, Sakamoto Y, Miller F, Glacial F, et al. Quantitative targeted absolute proteomic analysis of transporters, receptors and junction proteins for validation of human cerebral microvascular endothelial cell line hCMEC/D3 as a human blood-brain barrier model. Mol Pharm. 2013;10:289–96. 23137377 10.1021/mp3004308

[44] Cereijido M, Robbins ES, Dolan WJ, Rotunno CA, Sabatini DD. Polarized monolayers formed by epithelial cells on a permeable and translucent support. J Cell Biol. 1978;77:853–80. 567227 10.1083/jcb.77.3.853 PMC2110138

[45] Hellinger E, Veszelka S, Toth AE, Walter F, Kittel A, Bakk ML, et al. Comparison of brain capillary endothelial cell-based and epithelial (MDCK-MDR1, Caco-2, and VB-Caco-2) cell-based surrogate blood-brain barrier penetration models. Eur J Pharm Biopharm. 2012;82:340–51. 22906709 10.1016/j.ejpb.2012.07.020

[46] Wen J, Gao X, Zhang Q, et al. Optimization of tilmicosin-loaded nanostructured lipid carriers using orthogonal design for overcoming oral administration obstacle. Pharmaceutics. 2021;13:303. 33669090 10.3390/pharmaceutics13030303 PMC7996536

[47] Eigenmann DE, Xue G, Kim KS, Moses AV, Hamburger M, Oufir M. Comparative study of four immortalized human brain capillary endothelial cell lines, hCMEC/D3, hBMEC, TY10, and BB19, and optimization of culture conditions, for an in vitro blood-brain barrier model for drug permeability studies. Fluids Barriers CNS. 2013;10:33. 24262108 10.1186/2045-8118-10-33 PMC4176484

[48] Bernas MJ, Cardoso FL, Daley SK, Weinand ME, Campos AR, Ferreira AJ, et al. Establishment of primary cultures of human brain microvascular endothelial cells to provide an in vitro cellular model of the blood-brain barrier. Nat Protoc. 2010;5:1265–72. 20595955 10.1038/nprot.2010.76 PMC3109429

[49] Neal EH, Marinelli NA, Shi Y, McClatchey PM, Balotin KM, Gullett DR, et al. A simplified, fully defined differentiation scheme for producing blood-brain barrier endothelial cells from human iPSCs. Stem Cell Rep. 2019;12:1380–8. 10.1016/j.stemcr.2019.05.008 PMC6565873 31189096

[50] Qian T, Maguire SE, Canfield SG, Bao X, Olson WR, Shusta EV, et al. Directed differentiation of human pluripotent stem cells to blood-brain barrier endothelial cells. Sci Adv. 2017;3:e1701679. 29134197 10.1126/sciadv.1701679 PMC5677350

[51] Lu TM, Houghton S, Magdeldin T, Duran JGB, Minotti AP, Snead A, et al. Pluripotent stem cell-derived epithelium misidentified as brain microvascular endothelium requires ETS factors to acquire vascular fate. Proc Natl Acad Sci U S A. 2021;118:e2016950118. 33542154 10.1073/pnas.2016950118 PMC7923590

[52] Mina F, Comim CM, Dominguini D, Cassol OJ Jr., Igna D, Ferreira DM. Il1-beta involvement in cognitive impairment after sepsis. Mol Neurobiol. 2014;49:1069–76. 24234155 10.1007/s12035-013-8581-9

