# Supplementary material for: Novel paracellular marker based on ᴅ-dipeptide structure for highly sensitive quantification with UPLC-MS/MS for in vitro and in vivo blood-brain barrier permeability analysis
Source: Fluids Barriers CNS. 2026 Apr 10;23:57. doi: 10.1186/s12987-026-00806-5 (PMC13067629; doi:10.1186/s12987-026-00806-5)
Supplement: Supplementary file 1 — Supplementary Material 1: Table S1 parameters for the MS/MS detection of ᴅ-Tyr(Me)Orn and ᴅ-Tyr([2H3]Me)Orn in positive heated ESI and SRM. Table S2: Validation results for the ᴅ-Tyr(Me)Orn assay in the four different biological matrices. Table S3: IS normalized matrix effect and recovery. Figure S4: Representative chromatograms of study samples. Figure S5: Remaining A Lucifer Yellow and B fluorescein percentage of uptake solution (100 and 300 µM) in 96-well plates. Table S6: Statistical analysis of ᴅ-Tyr(Me)Orn Transwell® assays. Table S7: Statistical analysis of Lucifer Yellow and fluorescein Transwell® assays. [file 12987_2026_806_MOESM1_ESM.docx]

**Supplements**

**Novel paracellular marker based on ᴅ-dipeptide structure for highly sensitive quantification with UPLC-MS/MS for *in vitro* and *in vivo* blood-brain barrier permeability analysis**

Cindy Bay^1^, Eric Mühlberg^2,3^, Philipp Uhl^3^, Julia Carolin Stingl^1^, Gzona Bajraktari-Sylejmani^1^, Jürgen Burhenne^1^, Johanna Weiss^1,†, *^, Max Sauter^1,†^

**Table S1:** Parameters for the MS/MS detection of ᴅ-Tyr(Me)Orn and ᴅ-Tyr([^2^H_3_]Me)Orn in positive heated ESI and SRM.

| **Parameter** | ᴅ-Tyr(Me)Orn (ᴅ-Tyr([^2^H_3_]Me)Orn) |
| --- | --- |
| Capillary voltage  Cone voltage  Cone gas flow  Source temperature  Desolvation gas flow (N_2_)  Desolvation temperature  SRM transition [*m/*z]  Dwell time  Collision energy  Collision gas flow (Ar) | 0.5 kV  20 V  150 L/Hr  150 °C  1000 L/h  600 °C  310.2 → 115.0 (313.3 → 115.0)  47 ms  10 V  0.15 mL/min |

ESI: Electrospray ionization, SRM: Selected reaction monitoring

**Table S2:** Validation results for the ᴅ-Tyr(Me)Orn assay in the four different biological matrices.

| **Lysed cells** | | **LLOQ** | **Low QC** | **Mid QC** | **High QC** |
| --- | --- | --- | --- | --- | --- |
|  |  | **0.050 ng/mL** | **0.150 ng/mL** | **112.5 ng/mL** | **225 ng/mL** |
| **Intraday** | |  |  |  |  |
| **1** | Mean [ng/mL] | 0.0443 | 0.151 | 115 | 219 |
|  | Accuracy [%] / Precision [% CV] | 88.7/3.08 | 100.6/2.27 | 102.1/2.64 | 97.2/2.05 |
| **2** | Mean [ng/mL] | 0.0530 | 0.169 | 118 | 226 |
|  | Accuracy [%] / Precision [% CV] | 106.0/9.15 | 112.8/3.39 | 104.9/2.82 | 100.2/1.78 |
| **3** | Mean [ng/mL] | 0.0505 | 0.151 | 122 | 230 |
|  | Accuracy [%] / Precision [% CV] | 101.0/17.3 | 101.0/11.5 | 108.0/2.22 | 102.2/1.23 |
| **Interday** | |  |  |  |  |
|  | Mean [ng/mL] | 0.0489 | 0.156 | 118 | 225 |
|  | Accuracy [%] / Precision [% CV] | 97.7/12.9 | 103.8/ 8.56 | 105.0/3.39 | 99.9/2.65 |
| **Cell culture medium** | |  |  |  |  |
| **Intraday** | |  |  |  |  |
| **1** | Mean [ng/mL] | 0.0490 | 0.159 | 120 | 230 |
|  | Accuracy [%] / Precision [% CV] | 88.8/8.52 | 101.3/4.65 | 106.9/2.05 | 102.0/0.84 |
| **Mouse plasma** | | **LLOQ**  **0.100 ng/mL** | **Low QC**  **0.300 ng/mL** | **Mid QC**  **112.5 ng/mL** | **High QC**  **225 ng/mL** |
| **Intraday** | |  |  |  |  |
| **1** | Mean [ng/mL] | 0.111 | 0.284 | 113.2 | 216 |
|  | Accuracy [%] / Precision [% CV] | 110.6/5.26 | 94.8/2.90 | 100.6/1.16 | 96.1/2.58 |
| **Mouse brain** | |  | **LLOQ**  **0.300 ng/mL** |  |  |
| **Intraday** | |  |  |  |  |
| **1** | Mean [ng/mL] |  | 0.257 | 121 | 235 |
|  | Accuracy [%] / Precision [% CV] |  | 85.7/1.91 | 107.3/3.00 | 104.2/1.82 |

CV: Coefficient of variation; LLOQ: Lower limit of quantification; QC: Quality control.

N = 4 replicates at LLOQ and each QC concentration.

**Table S3:** IS normalized matrix effect and recovery.

| **Lysed cells** | **Mid QC** | **High QC** |
| --- | --- | --- |
|  | **112.5 ng/mL** | **225 ng/mL** |
| Matrix effect [%] | 94.4 | 94.4 |
| Recovery [%] | 100.4 | 99.4 |
| **Cell culture medium** | **Mid QC** | **High QC** |
|  | **112.5 ng/mL** | **22 ng/mL** |
| Matrix effect [%] | 97.5 | 92.7 |
| Recovery [%] | 101.3 | 104.5 |
| **Mouse plasma** | **Mid QC** | **High QC** |
|  | **112.5 ng/mL** | **225 ng/mL** |
| Matrix effect [%] | 93.0 | 110.1 |
| Recovery [%] | 118.2 | 113.5 |
| **Mouse brain** | **Mid QC** | **High QC** |
|  | **112.5 ng/mL** | **225 ng/mL** |
| Matrix effect [%] | 97.5 | 115.0 |
| Recovery [%] | 91.0 | 87.1 |

IS: internal Standard, QC: quality control. N = 3 replicates at each QC concentration.


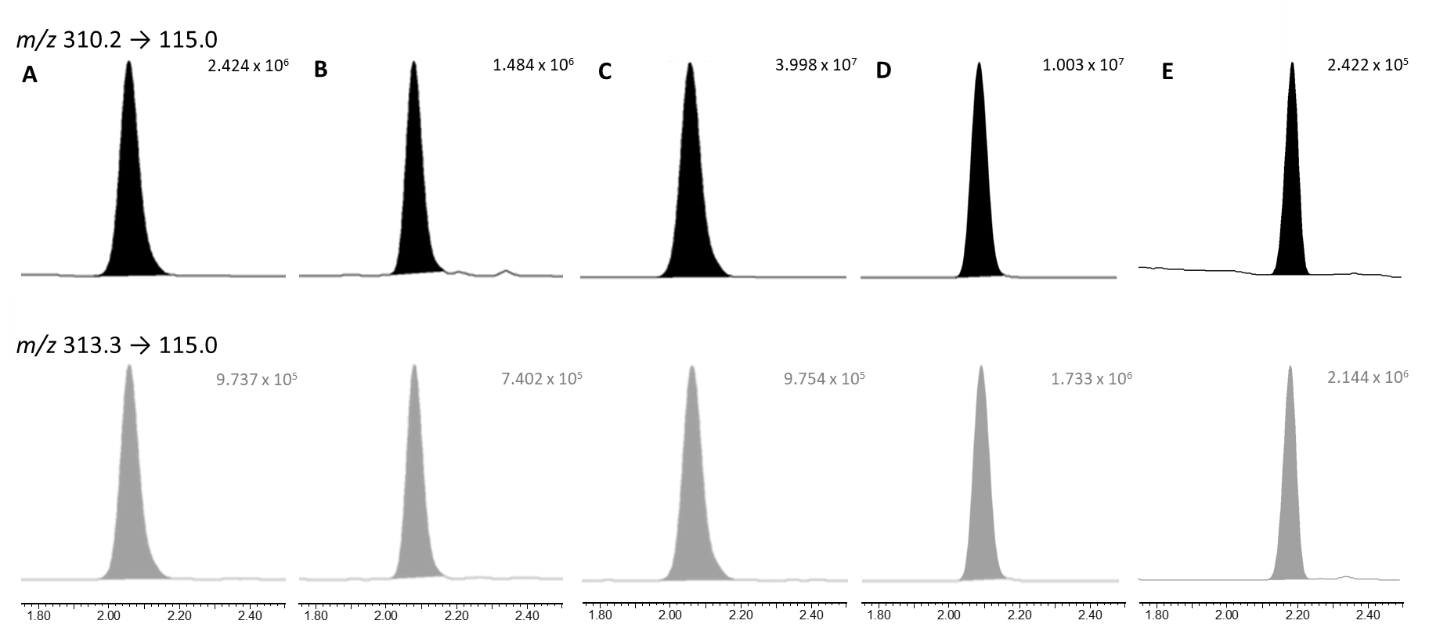


**Figure S4:** Representative chromatograms of study samples. In black the analyte is shown, in grey the internal standard. **A** liver homogenate sample, diluted 100 × in mouse plasma, concentration of 10.4 ng/mL; **B** brain homogenate, concentration of 6.83 ng/mL; **C** plasma sample, diluted 100 × in mouse plasma, concentration of 173 ng/mL; **D** Cell lysate sample, concentration of 17.8 ng/mL; **E** medium sample from Transwell^®^ experiments, diluted 20 × in medium, 0.648 ng/mL.

**Figure S5:** Remaining **A** Lucifer Yellow and **B** fluorescein depicted as percentage of uptake solution (100 and 300 µM) in 96-well plates.

**Table S6:** Statistical analysis of ᴅ-Tyr(Me)Orn Transwell^®^ assays.

|  | **MDCK** | | **hCMEC/D3** | | **iBCEC** | | **primary BCEC** | |
| --- | --- | --- | --- | --- | --- | --- | --- | --- |
| **10 min** |  | **p value** |  | **p value** |  | **p value** |  | **p value** |
| Cell monolayer vs. EGTA | ns | 0.0683 | ns | 0.9998 | ns | 0.8586 | ns | 0.7649 |
| Cell monolayer vs. empty wells | * | 0.0262 | ns | 0.8903 | ns | 0.3383 | * | 0.0457 |
| EGTA vs. empty wells | ns | 0.9123 | ns | 0.8742 | ns | 0.6514 | ns | 0.187 |
| **20 min** |  |  |  |  |  |  |  |  |
| Cell monolayer vs. EGTA | **** | <0.0001 | ns | 0.9023 | ns | 0.2839 | ns | 0.6625 |
| Cell monolayer vs. empty wells | **** | <0.0001 | ns | 0.9127 | * | 0.0418 | ** | 0.0022 |
| EGTA vs. empty wells | ns | 0.1255 | ns | 0.6631 | ns | 0.6299 | * | 0.024 |
| **30 min** |  |  |  |  |  |  |  |  |
| Cell monolayer vs. EGTA | **** | <0.0001 | ns | 0.8787 | ns | 0.0589 | ns | 0.5568 |
| Cell monolayer vs. empty wells | **** | <0.0001 | ns | 0.872 | *** | 0.0002 | **** | <0.0001 |
| EGTA vs. empty wells | ns | 0.1698 | ns | 0.5797 | ns | 0.1897 | ** | 0.002 |
| **40 min** |  |  |  |  |  |  |  |  |
| Cell monolayer vs. EGTA | **** | <0.0001 | ns | 0.4535 | ns | 0.1333 | ns | 0.1528 |
| Cell monolayer vs. empty wells | **** | <0.0001 | ns | 0.567 | **** | <0.0001 | **** | <0.0001 |
| EGTA vs. empty wells | ns | 0.1323 | ns | 0.0606 | *** | 0.0009 | **** | <0.0001 |
| **50 min** |  |  |  |  |  |  |  |  |
| Cell monolayer vs. EGTA | **** | <0.0001 | ns | 0.8042 | ns | 0.2453 | *** | 0.0002 |
| Cell monolayer vs. empty wells | **** | <0.0001 | ns | 0.87 | **** | <0.0001 | **** | <0.0001 |
| EGTA vs. empty wells | ** | 0.0041 | ns | 0.4747 | **** | <0.0001 | *** | 0.0008 |
| **60 min** |  |  |  |  |  |  |  |  |
| Cell monolayer vs. EGTA | **** | <0.0001 | ns | 0.9831 | * | 0.0237 | ** | 0.0029 |
| Cell monolayer vs. empty wells | **** | <0.0001 | ns | 0.3237 | **** | <0.0001 | **** | <0.0001 |
| EGTA vs. empty wells | *** | 0.0002 | ns | 0.3963 | *** | 0.0004 | **** | <0.0001 |

Statistical analysis was performed with ordinary two-way ANOVA with Turkey’s multiple comparison test, by comparing columns within each row (multiple comparison, each cell mean with every other mean).

**Table S7:** Statistical analysis of Lucifer Yellow and fluorescein Transwell^®^ assays.

|  | **Lucifer Yellow** | | | | **Fluorescein** | | | |
| --- | --- | --- | --- | --- | --- | --- | --- | --- |
|  | **MDCK** | | **hCMEC/D3** | | **MDCK** | | **hCMEC/D3** | |
| **10 min** |  | **p value** |  | **p value** |  | **p value** |  | **p value** |
| Cell monolayer vs. empty wells | * | 0.0037 | ns | 0.4040 | ns | 0.1148 | ns | 0.8758 |
| **20 min** |  |  |  |  |  |  |  |  |
| Cell monolayer vs. empty wells | **** | <0.0001 | ns | 0.2618 | ns | 0.0568 | ns | 0.5552 |
| **30 min** |  |  |  |  |  |  |  |  |
| Cell monolayer vs. empty wells | * | 0.0105 | ns | 0.4395 | ** | 0.0095 | ns | 0.3892 |
| **40 min** |  |  |  |  |  |  |  |  |
| Cell monolayer vs. empty wells | ** | 0.0017 | ns | 0.1017 | *** | 0.0002 | ns | 0.1050 |
| **50 min** |  |  |  |  |  |  |  |  |
| Cell monolayer vs. empty wells | ** | 0.0078 | * | 0.0375 | *** | 0.0003 | * | 0.0328 |
| **60 min** |  |  |  |  |  |  |  |  |
| Cell monolayer vs. empty wells | ** | 0.0015 | *** | 0.0009 | *** | 0.0003 | ** | 0.0053 |

Statistical analysis was performed with mixed-effects analysis with Geisser-Greenhouse correction and Šídák’s multiple comparison test, by comparing cell mean with the other cell mean in that row.
